# Optimizing national border reopening policies in the COVID-19 pandemic: A modeling study

**DOI:** 10.3389/fpubh.2022.979156

**Published:** 2022-11-30

**Authors:** Jiaoling Huang, Ying Qian, Wuzhi Shen, Yong Chen, Laijun Zhao, Siqi Cao, Eliot Rich, John Pastor Ansah, Fan Wu

**Affiliations:** ^1^School of Public Health, Shanghai Jiao Tong University School of Medicine, Shanghai, China; ^2^Business School, University of Shanghai for Science and Technology, Shanghai, China; ^3^Faculty of Social Sciences, University of Bergen, Bergen, Norway; ^4^Department of Profession Management, Shanghai Municipal Center for Disease Control and Prevention, Shanghai, China; ^5^School of Business, University at Albany, State University of New York, New York, NY, United States; ^6^Case Western Reserve University, Center for Community Health Integration, Duke-NUS Medical School, Singapore, Singapore; ^7^Shanghai Institute of Infectious Disease and Biosecurity, School of Public Health, Fudan University, Shanghai, China

**Keywords:** fever clinic unit monitoring, SEIR model, border reopening, vaccination, border screening

## Abstract

**Objective:**

After emergence of the COVID-19 pandemic and subsequent restrictions, countries worldwide have sought to reopen as quickly as possible. However, reopening involves the risk of epidemic rebound. In this study, we investigated the effective policy combination to ensure safe reopen.

**Methods:**

On the basis of the classical SEIR epidemic model, we constructed a COVID-19 system dynamics model, incorporating vaccination, border screening, and fever clinic unit monitoring policies. The case of China was used to validate the model and then to test policy combinations for safe reopening.

**Findings:**

Vaccination was found to be crucial for safe reopening. When the vaccination rate reached 60%, the daily number of newly confirmed COVID-19 cases began to drop significantly and stabilized around 1,400 [1/1,000,000]. The border screening policy alone only delayed epidemic spread for 8 days but did not reduce the number of infections. Fever clinic unit monitoring alone could reduce the peak of new confirmed cases by 44% when the case identification rate rose from 20 to 80%. When combining polices, once the vaccination rate reached 70%, daily new confirmed cases stabilized at 90 [0.64/1,000,000] with an 80% case identification rate at fever clinic units and border screening. For new variants, newly confirmed cases did not stabilize until the vaccination rate reached 90%.

**Conclusion:**

High vaccination rate is the base for reopening. Vaccination passport is less effective compared with a strong primary care monitoring system for early detection and isolation of the infected cases.

## Introduction

During the COVID-19 pandemic, countries have sought to reopen with fervent desires after long periods of border closure or restrictions so as to return to normalcy (1). According to a report released in 2021 by the International Organization for Migration and the Migration Policy Institute, travel measures and border closures peaked in mid-December 2020 when they were in force in more than 111,000 locations at one time (2). However, reopening involves multiple challenges, particularly with the continual emergence of new SARS-CoV-2 variants, the latest of which is the variant named Omicron. This situation leads to increased uncertainties regarding infection rates, the likelihood of severe illness, levels of vaccine protection, and treatment effectiveness. As the emergence of COVID-19 variants becomes a normal phenomenon, countries have gradually adapted and are exploring safe ways to reopen in the case of future restrictions.

Most countries are currently implementing vaccination campaigns before taking steps toward reopening. As of December 30, 2021, it is reported that an average of 49 doses of COVID-19 vaccine had been administered for every 100 people worldwide (3). Despite the controversies and uncertainties brought about by new variants, institutions and scholars generally believe that vaccines can effectively reduce the incidence of severe illness and death (4). Thus, a worldwide COVID-19 vaccine campaign is now underway across multiple countries (5, 6). The threat of the Omicron variant has even prompted countries to accelerate and broaden their roll-out of vaccine booster doses. Moreover, COVID-19 vaccine passports are also being discussed widely. Governments worldwide have put vaccine passports on the policy agenda, reflecting the desire to return to normalcy as the COVID-19 pandemic enters the next phase. The United States, United Kingdom, and European Union are currently considering the feasibility of such a policy measure; Australia, Denmark, and Sweden have committed to its implementation; and Israel is issuing “green passes” to vaccinated residents (7).

Vaccine passports offer one option in reopening as a border screening measure. However, opponents argue that even if vaccine passports are used, transmission of the virus cannot be completely stopped (8). Under such circumstances, monitoring patients suspected of having COVID-19 infection is indispensable. After the initial COVID-19 outbreak, monitoring policies based on primary healthcare systems were implemented in Singapore, Japan, the United Kingdom, and Canada (9–12). In China, a fever clinic monitoring system has operated in large hospitals since the SARS outbreak, and a program of fever clinic units was initiated at the beginning of the COVID-19 outbreak in 2020 (13). Distinguished from a fever clinic in a large hospital, fever clinic units are established at community health service centers, which are the primary care institutions in China. As an example, Shanghai has one of the earliest city-initiated fever clinic unit programs, with 225 units built so far covering nearly all community health service centers in Shanghai (14).

Reopening is a global issue after periods of restriction during the COVID-19 pandemic; however, reopening presents an enormous risk of epidemic rebound, especially with the added challenge of emerging variants. Most of current studies tried to figure out available policy choices and strategies for safe border reopening, simulate the possible risk of future outbreaks after reopening, examine the effectiveness of border control policies in combination with internal measures (15–19). And some studies further simulated a new plan replacing border restriction policy, contact tracing plan for instance (20). However, a series of forward-looking questions remains to be addressed: Is reopening feasible with vaccination? What vaccination rate is needed to ensure safe reopening? Can we achieve reopening and control of the epidemic using a combination of policies, especially under circumstances of new variants? Thus, we simulated the outcomes of multiple policy options, mainly including vaccination, border screening, and monitoring policies rather than quarantine policies, in a virtual environment in which real-world policy operations are restored. We aimed to determine which policy combination is optimal for next steps in reopening during the COVID-19 pandemic.

## Methods

### Model structure

On the basis of the classical SEIR (Susceptible–Exposed–Infectious–Recovered) epidemic model (21, 22), we established a system dynamics model for reopening in China during the COVID-19 pandemic, in two steps. First, we developed a base model for validating the COVID-19 transmission in China before reopening (simulation from 0 to 90 days). Second, we extended the base model incorporating reopening policies, including vaccination roll-out, border screening, and primary healthcare monitoring (simulation from 90 to 360 days). To better represent the real-world situation, we also considered the following factors in the reopening model: (1) vaccination effectiveness against infection, severe illness, and death; (2) waning of vaccination-induced immunity; and (3) the emergence of new variants.

### The base model

**Figure 1:**
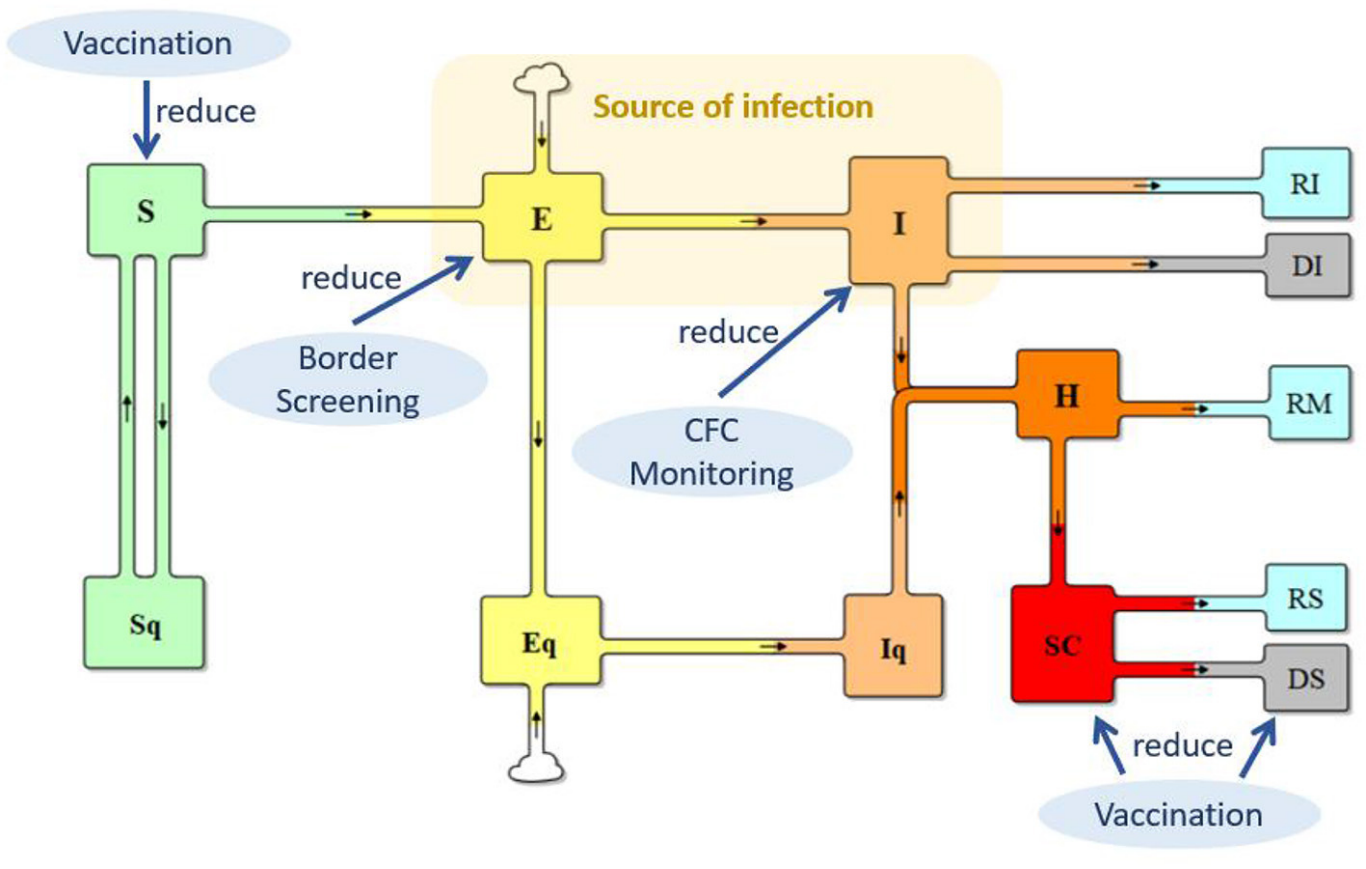
Extended SEIR model.

Built on the basis of the traditional SEIR model, the total population was differentiated into quarantined or not-quarantined groups, incorporating the quarantine policy implemented in China, as shown in Figure 1. *S* and *Sq* represent the unquarantined susceptible population and their counterparts in quarantine, respectively; (2) *E* and *Eq* represent unquarantined individuals without symptoms but have already got infected (people in incubation period), and their quarantined counterparts; (3) *I* and *Iq* represent symptomatic individuals with COVID-19 infection and their counterparts in quarantine; (4) H represents the hospitalized population; (5) SC represents the population of severe COVID-19 cases; (6) RI, RM, and RS represent the recovered from the I, H, and SC populations, respectively; and (7) DI and DS represent deaths from the I and SC.

The transmission from *S* to E reflects the infection process. In the case of COVID-19, individuals in both E and I can spread the virus when in contact with an individual in *S*, but E has a lower level of transmission probability. Supposing that the transmission probability of I is β, the transmission probability of E is θ ^*^ β (0 < θ < 1). With implementation of the isolation policy for suspected cases, μ in I are kept in isolation at fever clinic units. Thus, the source of infection is θ E + (1–μ) ^*^ *I*, and transmission from S to E can be calculated as (θE + (1–μ) ^*^ I) ^*^ *S*/*N*) ^*^ β ^*^ *c*, where c is the contact rate. The transmission from S to Sq represents the track–trace and quarantine of individuals in S, which can be represented by (1–μ) ^*^ *I* ^*^ S/N ^*^ (1–β) ^*^ *cq*, where q is the percentage of individuals who are tracked and quarantined. Track–trace and quarantine of those in *E* is ((1–μ) ^*^ I ^*^ S/N) ^*^ βcq. Therefore, the base model is as follows:


$$\frac{\text{dS}}{\text{dt}}=-\beta \text{c}((1-\mu )\text{I}+\theta \text{E})\text{S}/\text{N}-(1-\beta )\text{cq}(1-\mu )\text{IS}/\text{N}+\kappa {\text{S}}_{\text{q}}$$

$$\frac{\text{dSq}}{\text{dt}}=(1-\beta )\text{cq}(1-\mu )\text{IS}/\text{N}-\kappa {\text{S}}_{\text{q}}$$

$$\frac{\text{dE}}{\text{dt}}=\beta \text{c}(1-\text{q})(1-\mu )\text{IS}/\text{N}-\sigma \text{E }$$

$$\frac{\text{dEq}}{\text{dt}}=\beta \text{cq}(1-\mu )\text{IS}/\text{N}-\sigma {\text{E}}_{\text{q}}+\text{m}(\text{t})$$

$$\frac{\text{dI}}{dt}=\sigma \text{E}-\delta \text{I}-\alpha_{\text{I}}\text{I}-\text{​​}\gamma_{\text{I}}\text{I}$$

$$\frac{\text{dIq}}{\text{dt}}=\sigma {\text{E}}_{\text{q}}-\delta {\text{I}}_{\text{q}}$$

$$\frac{\text{dH}}{\text{dt}}=\delta {\text{I}}_{\text{q}}+\delta \text{I}-\omega (\delta {\text{I}}_{\text{q}}+\delta \text{I})-\gamma_{\text{H}}\text{H}$$

$$\frac{\text{dSC}}{\text{dt}}=\omega (\delta {\text{I}}_{\text{q}}+\delta \text{I})-\alpha_{\text{s}}\text{SC}-\gamma_{\text{s}}\text{SC}$$

$$\frac{dDI}{dt}=\alpha_{\text{I}}\text{I }$$

$$\frac{\text{dRI}}{\text{dt}}=\gamma_{\text{I}}\text{I }$$

$$\frac{\text{dDS}}{\text{dt}}=\alpha_{\text{s}}\text{SC}$$

$$\frac{\text{dRS}}{\text{dt}}=\gamma_{\text{s}}\text{SC}$$

$$\frac{\text{dRM}}{\text{dt}}=\gamma_{\text{H}}\text{H}$$

$$\begin{array}{l} \text{N}=\text{S}+{\text{S}}_{\text{q}}+\text{E}+{\text{E}}_{\text{q}}+\text{I}+{\text{I}}_{\text{q}}+\text{H}+\text{SC}+\text{RI}\\ \;+\text{RM}+\text{RS}+\text{DI}+\text{DS} \end{array}$$

where κ is the return rate from S_q_ to *S*; σ is the rate E or Eq develop symptoms; δ is the rate of hospital admission; ω is the proportion of severe cases; α_I_ and γ_I_ are the death rate and recovery rate of I without going to hospital; γ_H_ is the recovery rate of mild cases in hospital; and α_*s*_ and γ_s_ are the death rate and recovery rate of severe cases; m(t) is the inflow population during the incubation period. This base model is used for model validation, comparing the model simulation results with the historical data. A detailed explanation of the model equations is presented in the Supplementary material, section 1.1.

### The reopening model with combined policies

As shown in Figure 1, three policies, vaccination, border screening, and monitoring at fever clinic units, were applied to reduce *S, E*, and *I*, respectively, to slow SARS-CoV-2 transmission and COVID-19 infections. For vaccination, vaccination rate in China, the vaccination effectiveness against infection, severe illness, and death, and the waning effect of vaccination-induced immunity are included in the model. (For detailed information on vaccination, please see Supplementary material, section 2). Border screening policy considers the inflow population of international travelers, who are infected but have no symptoms. Policy of whether to require vaccination are considered. Monitoring at fever clinic units concerns the identification and isolation of suspected cases. The detailed changes in the model are summarized in Table 1.

**Table 1:** Variable changed in reopening model.

**Policies**	**Variable name**	**Base model**	**Reopening model**
Roll-out of vaccination	Susceptible population	*S*	S*(1-η*λ_1_(1-ν))
	Fraction of severe cases	ω	ω*(1–η*λ_2_(1–ν))
	Death fraction of severe cases	α_*I*_	ω*(1–η*λ_3_(1–ν))
Border screening	Inflow of E	Adding *m*(*t*) to *Eq*	Adding *m*(*t*)(1–$\lambda_{1}^{*}$ϕ_1_) to *E*
Fever clinic unit monitoring	Isolation of infected population with symptoms	(1–μ)*I	(1–μ)*I

Where η is the vaccination rate; λ_1_, λ_2_, λ_3_ are the vaccination effectiveness against infection, against severe cases and against death respectively; ν is the waning effect of vaccination-induced immunity; ϕ_1_ is a policy with 0 as no vaccination passport and 1 as applying vaccination passport. Note that fever clinic unit monitoring is one of the non-pharmaceutical interventions already implemented during the outbreak of COVID-19 in China. Therefore, no parameter change happened for this policy.

To address the challenge of new variants, we included a set of scenarios involving the Delta and Omicron variants. Owing to lack of data for Omicron, we simulated scenarios with optimistic, normal, and pessimistic parameter settings. Changes in the parameters included transmission possibility (β), proportion of severe cases (ω), and death fraction of severe cases (α_*I*_), and the effectiveness of vaccination against infection, against severe illness, and against death (λ_1_, λ_2_, λ_3_, respectively). Data and analysis of COVID-19 variants were discussed in the Supplementary material, section 3.

### Parameter setting

The definitions and settings of major parameters are described in Table 2, which include the value, the unit, and most importantly, the source information for parameter setting. Most sources are from previous literatures (23–28).

**Table 2:** Main parameter settings for reopening model.

**Variables**	**Value**	**Unit**	**Source and explanation**
β: Transmission probability	0.038	1/ times	Transmission probability is closely related to the basic reproduction number *R*0, which can be calculated as *R*0 = *βc*/σ. At the beginning of the COVID-19 outbreak in China, the R0 was estimated to be approximately 2.8 (23, 24). Therefore, β can be calculated to be 0.038.
σ: Transition rate	0.19	1/day	The reciprocal of the incubation period, which was on average 5.2 days (25, 26).
κ: The rate sq returning to *S*	1/14	1/day	The reciprocal of the duration of quarantine, which is 14 days in China (27).
θ: Infectiousness in incubation period	0.5	%	Infected patients in incubation period were with lower transmission probability (28).
*γ_*I*_*: Recovery rate for those not treated in hospitals	1/14	1/day	Individuals not treated in hospital were those had mild or no symptoms and would recover within 14 days on average (29).
*α_*I*_*: Death rate for those not treated in hospitals	0.002	1/day	Calibrated with data.
λ1: vaccination effectiveness against infectious	0.8	%	Based on data gathered from literature and news about vaccination, see Supplementary material, section 2.2.
λ2: vaccination effectiveness against severe cases	0.9	%	Based on data gathered from literature and news about vaccination, see Supplementary material, section 2.2.
λ3: vaccination effectiveness against death	0.8	%	Based on data gathered from literature and news about vaccination, see Supplementary material, section 2.2.

Other parameters such as the initial value of the population groups and the policy variables were presented the Supplementary material, section 1.2 and Table 2 respectively.

To address the challenge of new variants, we included a set of scenarios involving the Delta and Omicron variants. Owing to lack of data for Omicron, we simulated scenarios with optimistic, normal, and pessimistic parameter settings. Changes in the parameters included transmission possibility (β), proportion of severe cases (ω), and death rate (α_*I*_), and the effectiveness of vaccination against infection, severe illness, and death (λ_1_, λ_2_, λ_3_, respectively). Data and analysis of COVID-19 variants were discussed in the Supplementary material, section 3.

### Model validation

**Figure 2:**
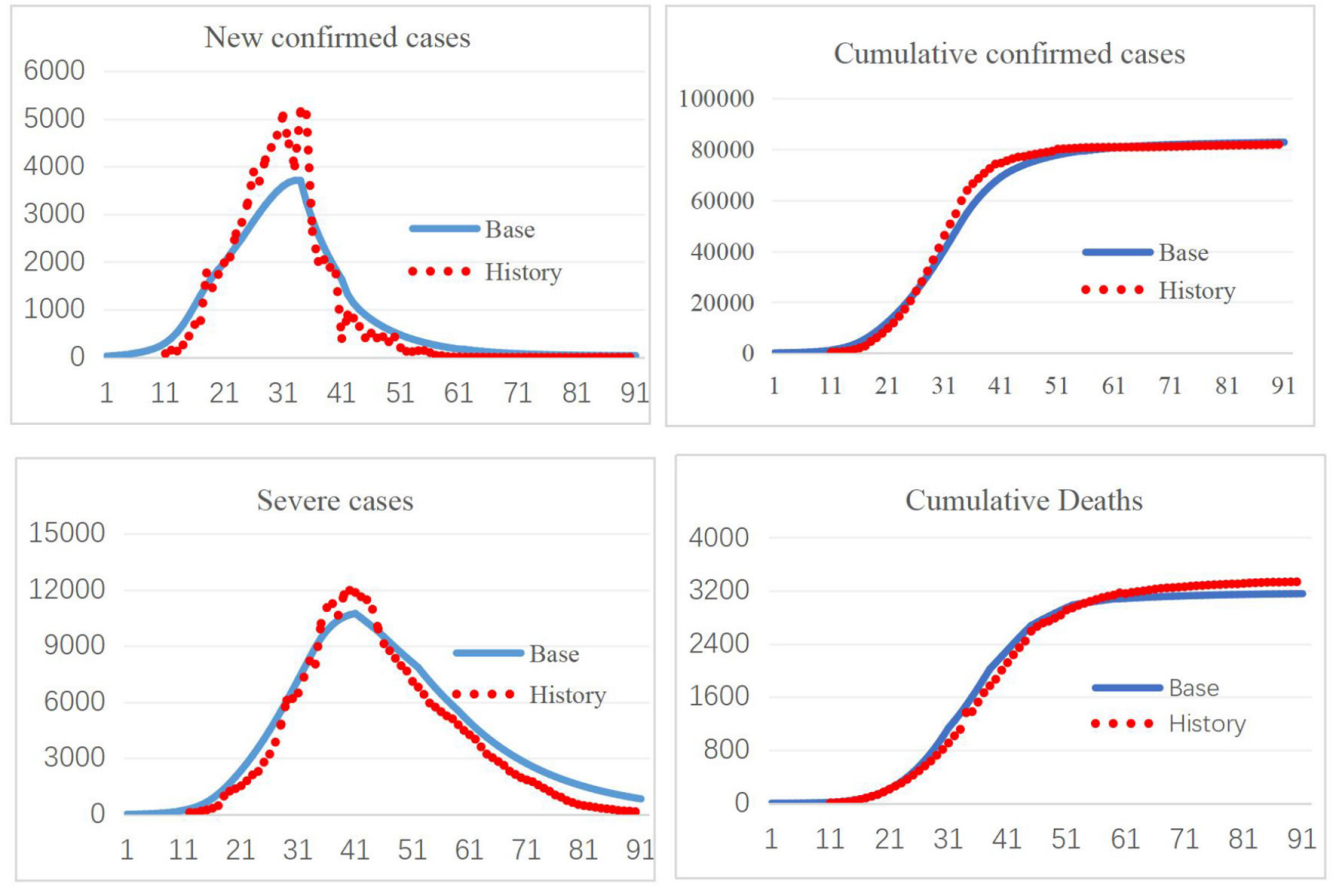
Simulation results and historical data.

The model was constructed in Vensim 8.09. Validation of a system dynamics model mainly includes verification of the model structure and model behavior under expected and extreme conditions. The SEIR model and its extension have been widely used to study the spread of infectious diseases, most recently in COVID-19 research, which illustrates the validity of the model structure (30, 31). Model behavior validation mainly compares the simulation results with real-world data. The first 3-month period corresponding to January 10–April 10, 2020, was used for model validation. As shown in Figure 2, the simulated new confirmed cases, cumulative confirmed cases, cumulative deaths, and cumulative recoveries fit well with the historical data, increasing confidence in the model. Further comparisons of the simulation results with historical data are provided in the Supplementary material, section 4.

## Results

Using our COVID-19 system dynamics model, we tested policies designed to facilitate reopening of national borders, first individually and then in combination. The simulations started from day 90 through day 360, assuming that policy changes occur at day 90.

### Policy scenario 1: Vaccination

**Figure 3:**
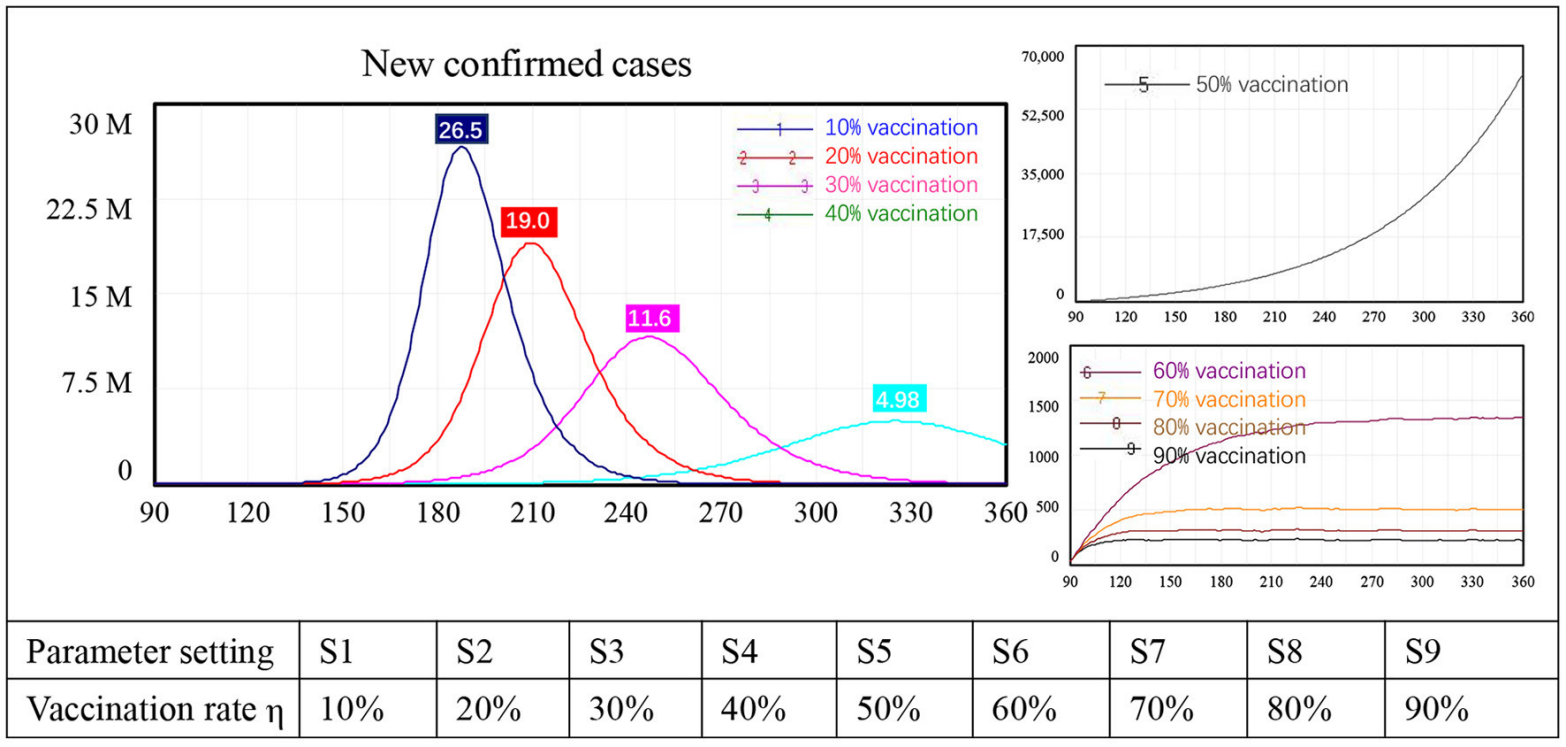
Simulation results for vaccination policy alone.

Because vaccination is being implemented in most countries, we first tested the impact of this intervention on border reopening. Changes in the vaccination rate from 10 to 90% with 10-percentage point intervals led to nine simulation results, numbered 1–9 in Figure 3.

Three types of behavior in terms of new confirmed cases were identified in the model. First, with low vaccination levels, the number of newly confirmed COVID-19 cases increased sharply after border reopening, peaking at approximately 26.5 million with a 10% vaccination rate, 19.0 million with 20% vaccination, 11.6 million with 30% vaccination, and 4.98 million with a 40% vaccination rate. Second, when the vaccination rate reached 50%, new confirmed cases still increased exponentially, but this increase was not as sharp as that seen in the first four scenarios. At the end of the simulation, the number of newly confirmed cases had not yet peaked, reaching approximately 61,000, which is unacceptably high. Third, when the vaccination rate was 60% or higher, the number of new confirmed cases stabilized instead of showing exponential growth. Specifically, case numbers stabilized at approximately 1,400 people with 60% of the population vaccinated, at around 500 with 70% vaccination, at 300 with 80% vaccination, and at 250 people with 90% vaccination. When the vaccination rate changed from 60 to 70%, newly confirmed cases dropped from 1,400 to 250, an 82% reduction. There was a diminishing impact on new confirmed COVID-19 cases after the vaccination rate reached 70%.

### Policy scenario 2: Border screening

Some countries now require international travelers to have a vaccination passport. Supposing that requiring vaccination for international travelers could reduce the inflow of infected people by 80%, we examined the impact of border entry screening when reopening borders. In this simulation, we assumed that our internal population had not been vaccinated whereas travelers had largely been vaccinated.

**Figure 4:**
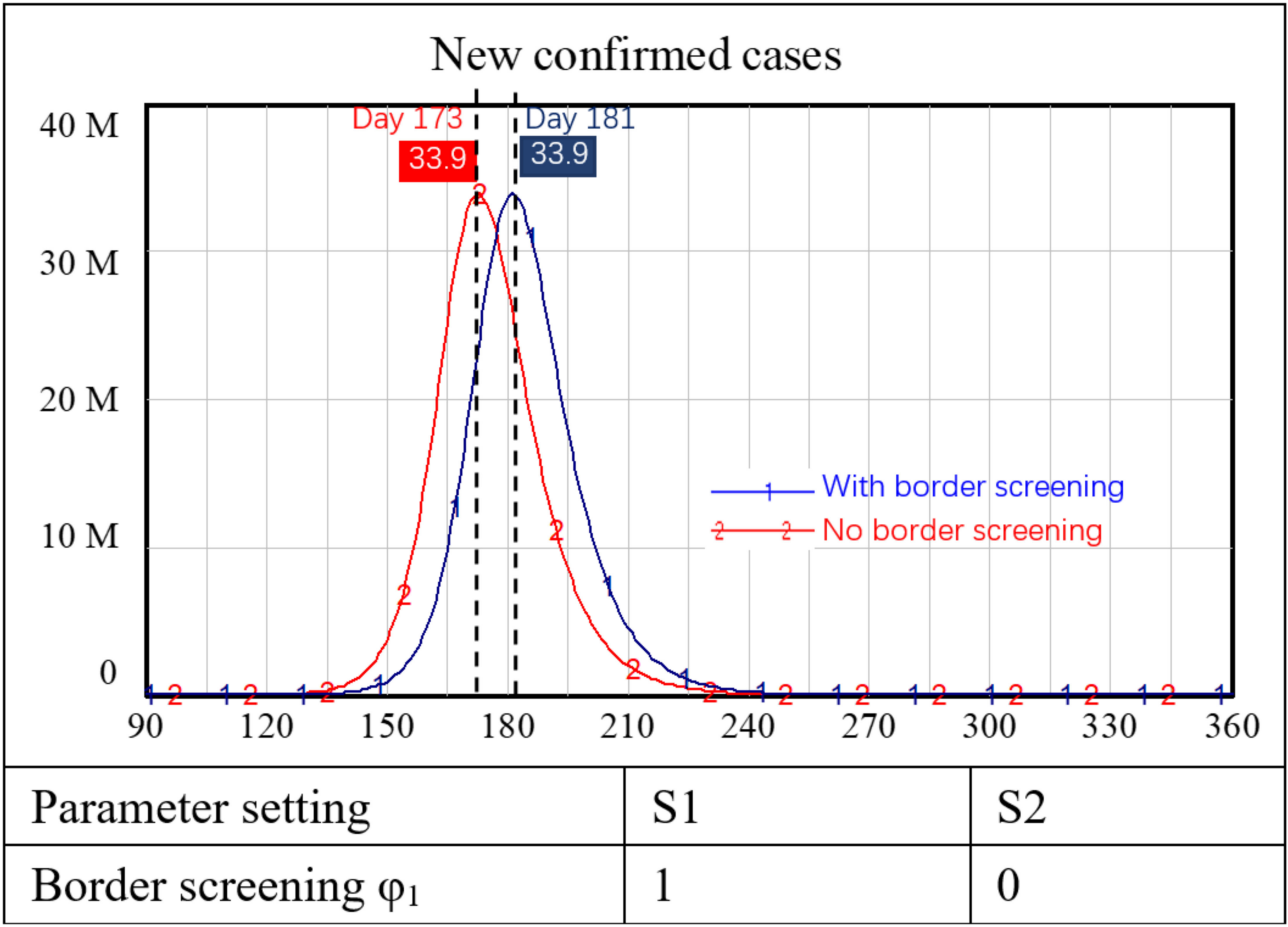
Simulation results for border screening policy alone.

The simulation results showed that with a border screening policy alone (i.e., no vaccination requirements or other prevention and control measures), the number of new confirmed cases of COVID-19 increased sharply. In the two scenarios with and without border screening, new confirmed cases both peaked at approximately 30 million people. The only difference was that the peak of new confirmed cases was delayed for 10 days with border screening, as shown in Figure 4.

### Policy scenario 3: Fever clinic unit monitoring

We found that the fever clinic unit monitoring policy to enable early identification of individuals with COVID-19 infection and rapid interruption of the chain of transmission was an effective policy in achieving safe reopening in China. Using the model, we tested scenarios where the case identification rate (IR) at fever clinic units reached 20, 40, 60, and 80% of ***I***, i.e., people who had developed symptoms of COVID-19. Again, these scenarios were considered separately from all other interventions.

The model simulation results showed that increasing the effectiveness of fever clinic units could reduce the peak number of newly confirmed cases. Specifically, fever clinic unit monitoring alone reduced the peak of new confirmed cases from 29.8 million to 16.7 million when the IR of fever clinic units increased from 20 to 80%. However, this policy alone cannot ensure safe border reopening. For the scenario where 80% of people with symptoms of COVID-19 (**I**) were identified at fever clinic units, cutting off routes of further infection, the number of new confirmed cases peaked at around 16.7 million 215 days after the reopening of borders, as shown in Table 3.

**Table 3:** Simulation results for fever clinic unit monitoring policy alone.

**Scenario 3** **fever** **clinic unit**				
	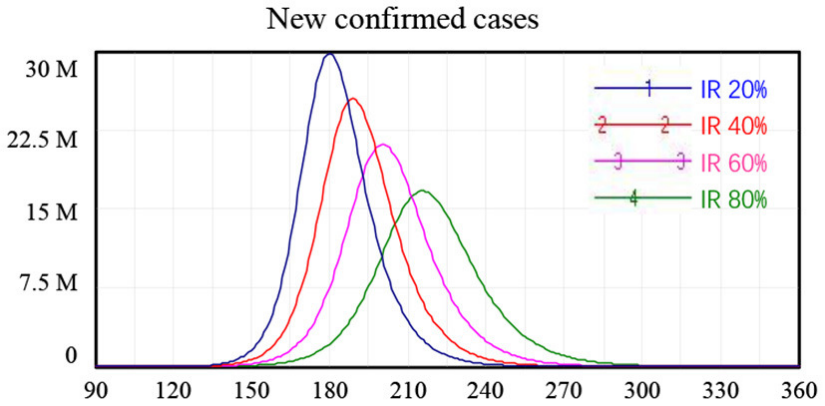		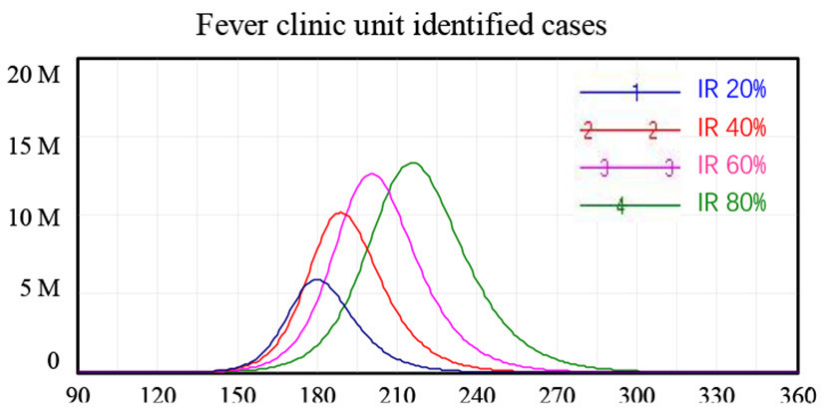	
**Parameter setting** ***μ***	**Peak value**	**Peak time**	**Peak value**	**Peak time**
S1: 20%	29.8 M	Day 180	5.9 M	Day 179
S2: 40%	25.5 M	Day 189	10.2 M	Day 189
S3: 60%	21.1 M	Day 200	12.6 M	Day 200
S4: 80%	16.7 M	Day 215	13.3 M	Day 216

*IR*, identification rate; *M*, million.

### Scenario 4: Combined policy simulations

**Figure 5:**
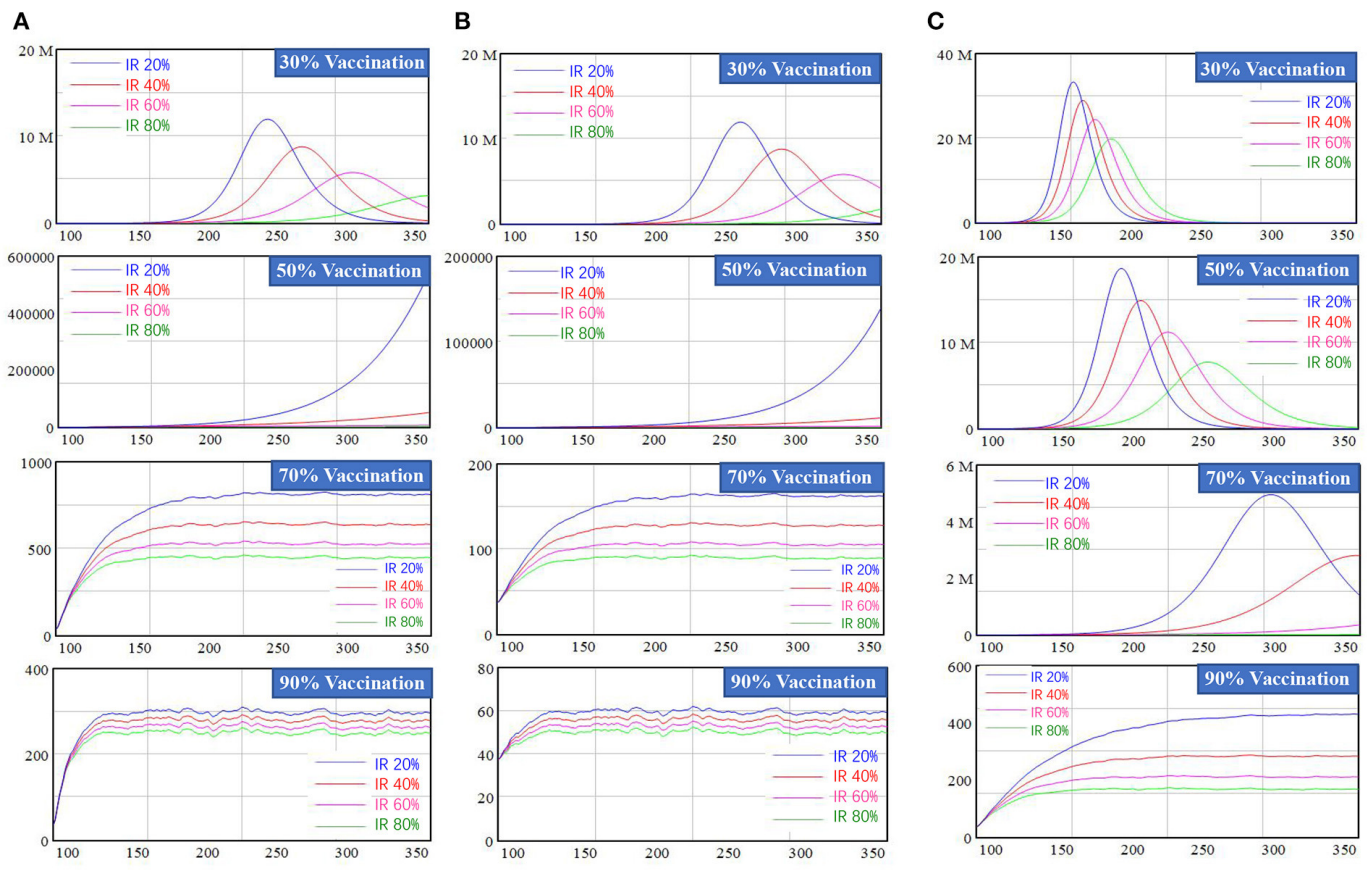
Simulation results of New confirmed cases for mixed policy combination. **(A)** Represents scenarios without border screening, **(B)** represents scenarios with border screening, and **(C)** represents the scenarios with a new variant with higher interactivity and lower vaccine effectiveness.

In this section, we examined the combined effect of the three policies: vaccination, border screening, and fever clinic unit monitoring. We simulated newly confirmed COVID-19 cases, severe cases, and deaths, among which the latter two showed results similar to those of the first scenario; therefore, we provide information of border screening and fever clinic unit monitoring policies in the Supplementary material, sections 6.1 and 6.2. Figure 5 presents the results regarding newly confirmed cases of COVID-19 infection, without border screening (a), with border screening (b), and the scenario of a new variant with 30% higher infectivity and lower vaccination effectiveness against infection, at 70% compared with 80% against the original SARS-CoV-2 strain (c).

In Figure 5, border screening was not applied in the first column. When the vaccination rate was less than 30%, new confirmed cases peaked at 3–12 million with high or low fever clinic unit IR rate, which is unacceptably high. Increasing the fever clinic unit IR rate under this scenario had a considerable impact: a 20% increase would mean a reduction of approximately 3 million newly confirmed cases people at the peak. When the vaccination rate reached 70%, new confirmed cases stabilized at 500 to 1,000 individuals, which is a relatively low level. Increasing the effectiveness of fever clinic units by 20% would lead to a drop in newly confirmed cases to approximately 100 persons. Border screening was added in the second column and only showed a delay effect when the vaccination rate was lower than 50%. Once the vaccination rate reached 70%, the number of new confirmed COVID-19 cases stabilized at a lower level, ranging from 90 to 160 persons. When considering waning vaccine-induced immunity, the number of new confirmed cases would rebound and a higher vaccination rate was required. Detailed results of simulation regarding this waning effect are provided in the Supplementary material, section 6.3.

The third column represents the new variant scenario, taking the widespread Delta virus as an example, in which the transmission probability was increased by 30% and the effectiveness of vaccination against infection declined from 80 to 70%. In this scenario, the number of new confirmed cases increased exponentially and peaked, even when the vaccination rate reached 50%. The number of new confirmed cases did not stabilize until the vaccination rate reached 90%. For example, when the vaccination rate was 90% and the IR of fever clinic units reached 80%, new confirmed COVID-19 cases stabilized at around 170 individuals, still higher than scenarios with the same variable settings but without considering a new variant. Vaccination effectiveness against different features of new variants and. We performed various scenarios using different SARS-CoV-2 variants, shown in the Supplementary material, section 6.4.

## Discussion

In the face of uncertainties that arise with the emergence of new variants, which has delayed the COVID-19 reopening process in many countries, we simulated the outcomes of reopening in a series of policy combinations covering multiple possibilities regarding the spread of new variants, effectiveness of vaccination against infection and severe disease, and decline of vaccine-induced immunity over time.

Undoubtedly, our study demonstrated that a robust vaccination rate is a prerequisite for reopening. The efficiency of the vaccine affects the simulation of the scenarios, including the number of new confirmed cases, hospitalized population, severe cases, deaths and recoveries. Taking new confirmed cases as an example, vaccination rates under 30% led to a rebound of COVID-19 after borders reopened, even with border screening and effective fever clinic unit monitoring in place. However, the marginal effect of higher vaccination rates diminished once the vaccination rate exceeded 70%, indicating that 70% is a critical threshold value. The effectiveness of vaccines against COVID-19 has been repeatedly verified and widely reported in numerous recent studies (32–34). However, vaccine effectiveness against the Delta variant gradually fell to 74.5–88% (after two doses) (6) and even to 39%, according to Israel's Ministry of Health (35). Lower effectiveness requires a higher vaccination rate to ensure safe reopening. According to our reopening model, the number of newly confirmed cases did not stabilize until the vaccination rate reached 90% in the scenario of a new variant. Similarly, Anderson and colleagues estimated that a vaccination rate of 75–90% would be necessary to achieve herd immunity if the proportional vaccine efficacy was considered (36). According to our study findings, implementing vaccination policies alone is not optimal with respect to safe reopening.

Border screening has been adopted by most countries to avoid the entry of infected people, to the extent possible. However, we found that the effect of vaccination passports was quite limited, which alone only served to delay infection for 8 days under our simulated scenarios. Understandably, merely keeping infected individuals away rather than implementing intervention measures would not result in a reduction in the local number of infected cases. Additionally, the ongoing identification of new COVID-19 cases has consistently shown that vaccines cannot fully prevent the occurrence of infection, e.g., in the case of the Delta, fully vaccinated individuals are reported to be infected (37). Therefore, proof of vaccination does not guarantee absolute safety. Such an idea was echoed in one modeling study by Rahmandad and colleagues who pointed out that effective responses to the COVID-19 pandemic require integrating factors, especially regarding early identification of new infections (38).

Notably, the effectiveness of fever clinic units was considerable in curbing the spread of infection before the COVID-19 vaccination rate reached a high level, reducing the peak of confirmed cases. Different from fever clinics in large hospitals, these units are located within China's densely distributed primary healthcare institutions, which are very close to or right in residential communities. Local qualitative studies have revealed that fever clinic units are an effective and economical solution to risk identification, embedded within primary healthcare institutions and with low construction and operation costs (39). According to the experience of China and our simulation model, fever clinic units are worthy of greater attention as an economical and effective approach against COVID-19.

According to our study, combined policies could achieve a comparatively optimized outcome, the idea of which has been widely supported (40, 41). Border screening via vaccine passport in the reopening model, together with vaccine and monitoring policies, would have an impact on the number of imported COVID-19 infections. Vaccine and primary care monitoring policies are complementary, according to our reopening simulation. If the vaccination rate is less than 70%, the effectiveness of monitoring should be maintained at a high level, otherwise, the monitoring effectiveness could be lower than 40%. We noticed that the daily number of newly confirmed cases stabilized at around 120 with an IR in fever clinic units of 40% and a 70% vaccination rate. However, the screening rate at fever clinic units must be consistently maintained at a high level (at least 60%) because vaccine-induced immunity will decline over time. Additionally, the effect of fever clinic units decreases as the vaccination rate increases to a high level and the epidemic is brought under control, according to our modeling results. Thus, the importance of fever clinic units may easily be forgotten once an epidemic has ended. We highlight the importance of maintaining this type of primary care monitoring system. In the face of challenges brought about by the emergence of new SARS-CoV-2 variants, we also advocate personal protection measures, such as mask wearing and social distancing, in addition to monitoring at fever clinic units. As Skegg et al. have pointed out, it could be catastrophic if such measures were relaxed prematurely during the ongoing pandemic (42).

## Implications

Using the case of China, we have provided evidence-based solutions to the global problem of reopening during the COVID-19 pandemic. Vaccination was proved to be a critical intervention for reopening in this study, with a 70–90% vaccination rate needed to ensure safe reopening. In areas where vaccines are still in short supply, we suggest that most susceptible populations should be given priority for vaccination to reduce the occurrence of severe disease and death. Monitoring suspected cases in fever clinic units within the primary healthcare system could be effective in controlling the spread of a post-reopening epidemic in the very early stages. In the scenarios of emerging variants with higher infectivity and lower vaccination effectiveness against infection, fever clinic unit monitoring is even more critical in controlling an epidemic wave. We also encourage governments around the world to facilitate the monitoring function of the primary healthcare system, such as in a fever clinic unit, which is crucial at the reopening stage. Most importantly, we recommend combined policies to achieve optimal outcomes against COVID-19, with at least 70% vaccination rate as threshold, supplemented by effective primary care monitoring system with at least 60% identification rate for safe border reopening.

## Strength and limitations

This study systematically simulated multiple border reopening scenarios focusing on new confirmed cases, severe cases, and cumulative deaths, and tried to figure out a safe reopening strategy by testing multiple policy choices responding to reopening risk. However, unexpected emergency situations of COVID-19 could hardly be predicted in current models, though new variants of COVID-19 had been soundly considered.

## Data availability statement

The original contributions presented in the study are included in the article/Supplementary material, further inquiries can be directed to the corresponding authors.

## Author contributions

YQ, JH, and FW managed this study, including formulation the research goals and aims, and management. JH and YQ did formal analysis and including mathematical or modeling and wrote the original draft. JH and FW acquired fundings. YQ, WS, LZ, and SC developed or designed the methodology date and including creation of models. YQ, WS, and SC collected date and including scrub data and maintain research data. YQ, JH, LZ, ER, JP, and FW reviewed and edited the manuscript, including giving critical, review, and commentary or revision. All authors contributed to the article and approved the submitted version.

## Funding

This study was supported by Shanghai Municipal Health Commission (Grant GWV-13 to FW), the Science and Technology Committee of Shanghai Municipality (Grants 22692192300 and 21692190200 to JH), the Science and Technology Innovation Project of Shanghai Jiao Tong University School of Medicine-Humanities and Social Sciences (Grant WK2102 to JH), and Shanghai Jiao Tong University China Hospital Development Institute 2022 Hospital Management Project (Grant CHDI-2022-B-31 to JH). The funders had no role in the design, data collection, analysis, interpretation, or writing of the report.

## Conflict of interest

The authors declare that the research was conducted in the absence of any commercial or financial relationships that could be construed as a potential conflict of interest.

## Publisher's note

All claims expressed in this article are solely those of the authors and do not necessarily represent those of their affiliated organizations, or those of the publisher, the editors and the reviewers. Any product that may be evaluated in this article, or claim that may be made by its manufacturer, is not guaranteed or endorsed by the publisher.
