# Supplementary material for: Optimizing national border reopening policies in the COVID-19 pandemic: A modeling study
Source: Front Public Health. 2022 Nov 30;10:979156. doi: 10.3389/fpubh.2022.979156 (PMC9749815; doi:10.3389/fpubh.2022.979156)
Supplement: Supplementary file 1 [file Data_Sheet_1.docx]

**Supplementary appendix**

Table of Content

[1 Model Description and Definitions 2](#_Toc92315671)

[1.1 Model structure 2](#_Toc92315672)

[1.1.1 The base model 2](#_Toc92315673)

[.1.2 The reopening model 5](#_Toc92315674)

[1.2 Parameter setting 6](#_Toc92315675)

[1.2.1 The initial value of the population groups 6](#_Toc92315676)

[1.2.2 Variables related to the feature of corona virus 7](#_Toc92315677)

[1.2.3 The policy control variables 8](#_Toc92315678)

[2 Vaccination 10](#_Toc92315679)

[2.1 Vaccine roll out 10](#_Toc92315680)

[2.2 Vaccine effectiveness 12](#_Toc92315681)

[2.3 Waning of vaccine-induced immunity 13](#_Toc92315682)

[3 The COVID-19 Variants 13](#_Toc92315683)

[3.1 Transmissibility 13](#_Toc92315684)

[3.2 Severe illness 14](#_Toc92315685)

[3.3 Vaccine effectiveness 14](#_Toc92315686)

[4 Model validation 14](#_Toc92315687)

[4.1 Data 14](#_Toc92315688)

[4.2 Simulation results and historical data 15](#_Toc92315689)

[4.3 Sensitive tests 16](#_Toc92315690)

[5 Forward projection 19](#_Toc92315691)

[5.1 Severe cases under combined policy 19](#_Toc92315692)

[5.2 Cumulative deaths under combined policy 20](#_Toc92315693)

[5.3 Waning effects of vaccine-induced immunity 21](#_Toc92315694)

[5.4 COVID-19 variants scenarios 22](#_Toc92315695)

[Reference 24](#_Toc92315696)

# Model Description and Definitions

In this section, we provide a detailed description of our model, including model structure, equations, and parameter setting procedures.

## Model structure

### 1.1.1 The base model

The Chinese government activated the first-level emergency response mechanism soon after the outbreak of COVID-19 and four types of non-pharmaceutical interventions were adopted:

I: Reduce contact, including lockdown epidemic areas, restrictions of traffic, banning social gatherings, and closure of workplaces, schools, and entertainment venues.

II: Quarantine high-risk individuals, including close contacts of confirmed or suspected cases, international arrivals, and people with a recent history of staying in epidemic areas. Centralized quarantine or home medical observation were taken out according to the risk of exposure.

III: Isolate suspected cases, enhancing fever clinics at designated hospitals, and establishing fever clinics at the primary healthcare, located in neighbourhood communities. Fever clinic is separated hospitals unit which can receive suspect patients at any time and well-equipped to reduce cross-infection risks.

IV: Reduce severe cases and death, including expert consultation, updating treatment guidelines, and providing sufficient medical resources. Early identification and early treatment were also ways to improve medical treatment.

Based on these four types of measures, we set up related policy control variables, which were contact rate **c**, quarantine rate **q**, isolation rate **u**, and hospital acceptance rate **δ**, fraction of severe case **ω**, the recovery rate of mild and severe cases **γ_H_**, **γ_S_** and death rate of severe cases **α_S_**. The total population of the country is **N**, which was stratified into the following categories, as shown in Figure 1.


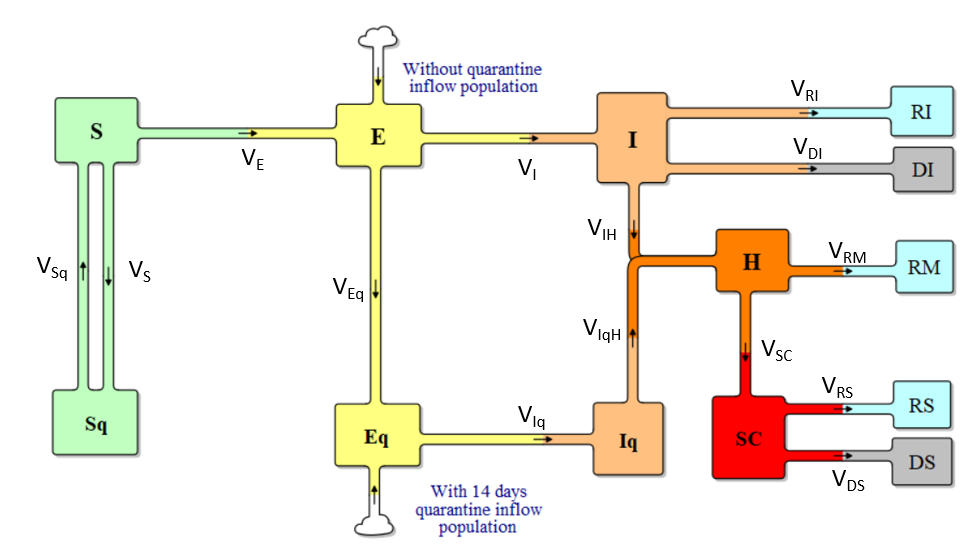
Figure 1 The base model for COVID-19 transmission in China

1. **S** represented the susceptible population, and **Sq** reflected those in quarantine;
2. **E** represented the infected population in the incubation period, and **Eq** reflected those in quarantine;
3. **I** represented the infected population with symptoms, and **Iq** reflected those in quarantine;
4. **H** represented the population accepted in a hospital; **SC** represented the population developed into severe cases. **RM** represented the recovered populations from mild cases; **RS** represented the recovered population from severe cases; **DS** represented the died population from severe cases.
5. **RI** represented the recovered population without treatments; **DI** represented the died population without treatments.

In the case of COVID-19, **E** could also transmit the disease when contacting with **S**, which was similar to **I**, but at a lower level of transmission probability. Set the transmission probability of **I** as β, the transmission probability of **E** is θ•β (0<θ<1). Following the isolation policy, part of **I** were isolated at fever clinic unit. Assume u percent of **I** are isolated, then the effective infection sources was θ•E+(1-μ)•I. The spread of disease happened when effective infection sources contact **S**, so the number of newly infected people was (θ•E+(1-μ)•I)•S/N•c•β, where **c** is the contact rate. Following the quarantine policy, part of the close contact of **I** were tracked and quarantined (Note that as E were people in incubation period, they were not identified and their close contacts were not traced). Let q percent of close contact being quarantined, then the number of **E** quarantined was (1-μ)•I•S/N•c•β•q and the number of **S** quarantined was (1-μ)•I•S/N•c•(1-β)•q, thus

V_E_ = (θ•E+(1-μ)•I)•S/N•c•β

V_Eq_= (1-μ)•I•S/N•c•β•q

(1)

V_S_ = (1-μ)•I•S/N•c•(1-β)•q

**E** and **Eq** would develop symptoms after the incubation period, becoming **I** and **Iq** respectively at a rate of σ, where σ is the reciprocal of the incubation period, thus

V_I_= E • σ

(2)

V_Iq_= Eq • σ

**I** and **Iq** were admitted to hospital at the rate δ where δ was the reciprocal of the waiting time to be accepted by the hospital. The hospitalization waiting time depended on the adequacy of medical resources, such as testing capacity and hospital bed availability.

V_IH_=δ • I

(3)

V_IqH_= δ•Iq

Meanwhile, **I** might recover or die without hospital treatment. Assume the self-recover rate was γ_I_ and the death rate is α_I_, thus:

V_RI_= I•γ_I_

(4)

V_DI_=I•α_I_

Some portion of the infected cases accepted into the hospital would develop into severe cases. Assume the fraction of severe cases is ω, thus:

(5)

V_SC_= ω•(δ • I + δ • Iq)

Severe cases transfer from **H** to **SC** and mild cases remained in H. Assume the recover rate of mild case was γ_H_, the recover rate of severe case was γ_S_, and the death rate of severe case was α _S_, thus:

V_RM_= H • γ_H_

(6)

V_RS_= SC • γ_S_

V_DS_= SC • α_S_

Based on these, the differential equations of the COVID-19 China model could be derived as follows:

$$\begin{matrix} \frac{\text{dS}}{\text{dt}}=-\text{βc((1}-\text{μ)I}+\text{θE)S/N}-\text{(1}-\text{β)cq(1}-\text{μ)IS/N}+\text{κS}_{\text{q}} \\ \frac{\text{dSq}}{\text{dt}}=\text{(1}-\text{β)cq(1}-\text{μ)IS/N}-\text{κS}_{\text{q}} \\ \frac{\text{dE}}{\text{dt}}=\text{βc(1}-\text{q)(1}-\text{μ)IS/N}-\text{σE} \\ \frac{\text{dEq}}{\text{dt}}=\text{βcq(1}-\text{μ)IS/N}-\text{σE}_{\text{q}}+\text{m(t)} \\ \frac{\text{dI}}{\text{dt}}=\text{σE}-\text{δI}-\text{α}_{\text{I}}\text{I}-\text{γ}_{\text{I}}\text{I} \\ \frac{\text{dIq}}{\text{dt}}=\text{σE}_{\text{q}}-\text{δI}_{\text{q}} \\ \frac{\text{dH}}{\text{dt}}=\text{δI}_{\text{q}}+\text{δI}-\text{ω(δI}_{\text{q}}+\text{δI)}-\gamma_{\text{H}}\text{H} \\ \frac{\text{dSC}}{\text{dt}}=\text{ω(δI}_{\text{q}}+\text{δI)}-\text{α}_{\text{S}}\text{SC}-\text{γ}_{\text{S}}\text{SC} \\ \frac{\text{dDI}}{\text{dt}}=\text{α}_{\text{I}}\text{I} \\ \frac{\text{dRI}}{\text{dt}}=\text{γ}_{\text{I}}\text{I} \\ \frac{\text{dDS}}{\text{dt}}=\text{α}_{\text{s}}\text{SC} \\ \frac{\text{dRS}}{\text{dt}}=\text{γ}_{\text{S}}\text{SC} \\ \frac{\text{dRM}}{\text{dt}}=\text{γ}_{\text{H}}\text{H} \\ \text{N}=\text{S}+\text{S}_{\text{q}}+\text{E}+\text{E}_{\text{q}}+\text{I}+\text{I}_{\text{q}}+\text{H}+\text{SC}+\text{RI}+\text{RM}+\text{RS}+\text{DI}+\text{DS} \end{matrix}$$

### 1.2 The reopening model

Three policies, vaccination, border screening, and fever clinic unit monitoring were applied to reduce S, E and I respectively, thus slowing down the transmission of the epidemic, as shown in Figure 2. Therefore, compared to the base model, some variables related to the reopening policies were added. Table 1 summarised the changes happened from base model to reopening model.


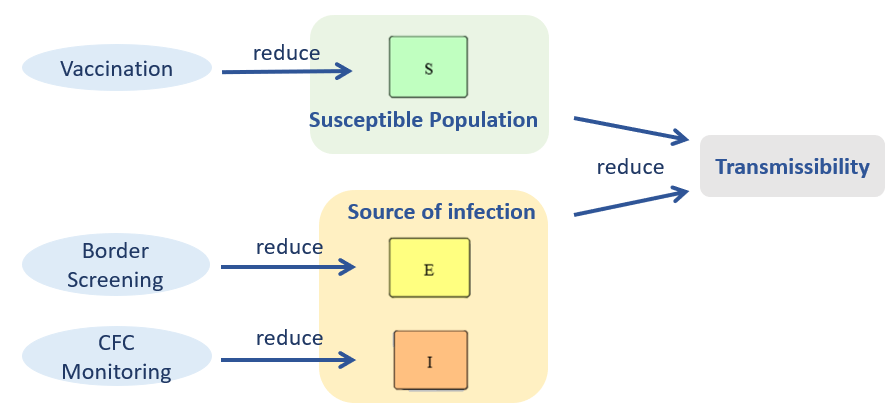


Figure 2 The reopening model with three policies

Table 1 Variable changed in reopening model

| Policies | Variable name | Base model | Reopening model |
| --- | --- | --- | --- |
| Roll-out of vaccination | Susceptible population | S | S*(1-η*λ_1_(1-ν)) |
|  | Fraction of severe cases | ω | ω*(1-η*λ_2_(1-ν)) |
|  | Death rate of severe cases | $\alpha_{I}$ | $\alpha_{I}$*(1-η*λ_3_(1-ν)) |
| Border screening | Inflow of E | m(t) | m(t)(1-λ_1_*φ_1_) |
| Fever clinic unit monitoring | Isolation of infected population with symptoms | (1- μ)*I | (1- μ)*I |

where η is the vaccination rate; λ_1_, λ_2_, λ_3_ are the vaccination effectiveness against infection, severe cases and death respectively; ν is the waning effect of vaccination-induced immunity; and φ_1_ is a policy with 0 as no vaccination passport and 1 as applying vaccination passport. Note that fever clinic unit monitoring is one of the non-pharmaceutical interventions already implemented during the outbreak of COVID-19 in China. Therefore, no parameter change happened for this policy.

## Parameter setting

Three types of parameters required value setting: 1) the initial value of the population groups; 2) the scenario setting variables that remained constant in one scenario and could change in different scenarios; 3) policy control variables that changed over time in one scenario due to the gradual implementation of policies.

### 1.2.1 The initial value of the population groups

The initial value of the population groups was mostly set based on the data published by the national health commission of the People’s Republic of China. Some of them were estimated based on the epidemic data. Detailed information were provided in Table 2.

Table 2 The initial value of population groups

| **Population group** | **Value** | **Unit** | **Source and Explanation** |
| --- | --- | --- | --- |
| S(0): Susceptible population not in quarantine | 1.4*10^9^-1330 | person | Calculation based on the equation of total population. |
| Sq(0): Susceptible population in quarantine | 739 | person | Epidemic data released. ^2^ |
| E(0): Infected people during incubation period but not in quarantine | 450 | person | The incubation period is 5.2 days and the initial waiting time to be accepted in hospital is about 4 days. Therefore, the initial level is referred to the new confirmed cases around day 9. |
| Eq(0): Infected people during incubation period in quarantine | 0 | person | No quarantine policy was implemented at the beginning of the epidemic. |
| I(0): Infected people with symptoms but not in quarantine | 100 | person | The initial waiting time to be accepted in hospital is about 4 days. Therefore, the initial level is referred to the new confirmed cases around day 4. |
| Iq(0): Infected people with symptoms in quarantine | 0 | person | No quarantine policy was implemented at the beginning of the epidemic. |
| H(0): The hospitalised population | 28 | person | Epidemic data released.^2^ Total hospitalized population is 38. In the beginning, due to limited knowledge of how to treat COVID-19, many cases developed into severe cases. |
| SC(0): The severe cases | 10 | person |  |
| RM(0): The recovered population from hospital | 2 | person | Epidemic data released.^2^ |
| RI(0): The recovered population without hospital treatment | 0 | person | No self-recovered at the beginning of the epidemic. |
| RS(0): The recovered population from severe cases | 0 | person | No recovered people from severe cases at the beginning of the epidemic. |
| DS(0): Deaths from severe cases | 1 | person | Epidemic data released. ^2^ |
| DI(0): Deaths from infected population without hospital treatment | 0 | person | There were no non-hospital deaths at the beginning of the epidemic. |
| N: Total population | 1.4*109 | person | Demographic data of the Bureau of Statistics.^1^ |
| m: inflow infected population | 150 | Person /day | The average inflow infected population in 18, the current number of international travelers is around 1/8 of normal condition without COVID-19. Therefore, it is estimated that when border reopens, the inflow of infected population would be around 150 person per day. |

### 1.2.2 Variables related to the feature of corona virus

The scenario setting variables are mostly related to the feature of the corona virus, such as transmission probability, β, the incubation period 1/ σ, the infectiousness in incubation period, θ, and the recover rate and death rate without hospital treatment γ_I_ and α_I_. New variant scenarios would change the value of one or more of these variables. The setting of base scenario were provided in Table 3.

Table 3 Variable settings for corona virus

| **Variables** | **Value** | **Unit** | **Source and Explanation** |
| --- | --- | --- | --- |
| β: Transmission probability | 0.038 | 1/ times | Transmission probability is closely related to the basic reproduction number R0, which can be calculated as R0=βc/σ. At the beginning of the COVID-19 outbreak in China, the R0 was estimated to be approximately 2.8.^3, 4^ Therefore, β can be calculated to be 0.038. |
| σ: Transition rate | 0.19 | 1/day | The reciprocal of the incubation period, which was on average 5.2 days.^5, 6^ |
| κ: The rate Sq returning to S | 1/14 | 1/day | The reciprocal of the duration of quarantine, which is 14 days in China. ^7^ |
| θ: Infectiousness in incubation period | 0.5 | % | Infected patients in incubation period were with lower transmission probability.^8^ |
| γ_I_: Recovery rate for those not treated in hospitals | 1/14 | 1/day | Individuals not treated in hospital were those had mild or no symptoms and would recover within 14 days on average.^9^ |
| α_I,_: Death rate for those not treated in hospitals | 0.002 | 1/day | Calibrated with data. |
| λ_1_: vaccination effectiveness against infectious | 0.8 | % | See section 2.2 below |
| λ_2_: vaccination effectiveness against severe cases | 0.9 | % | See section 2.2 below |
| λ_3_: vaccination effectiveness against death | 0.8 | % | See section 2.2 below |
| φ_1_: Quarantining immigrants or not | 0, 1 | dmnl | Policy variable, where 0 reflected without vaccination passport and 1 reflected with vaccination passport. |

### 1.2.3 The policy control variables

Policy control variables are those related to the non-pharmaceutical interventions implemented in China, such as the contact rate c, the quarantine rate q, the identification rate μ, the waiting time for acceptance to hospital 1/ δ, recover rate of mild cases and severe cases γ_H_, γ_S_, and the death rate of severe cases are variables. These variables changed over time as the gradual implementation of the policies. The basic setting of the control variables were shown in Table 4.

Table 4 Policy control variables and their setting basis

| **Policy variable settings** | **Setting basis** |
| --- | --- |
| c: Contact rate  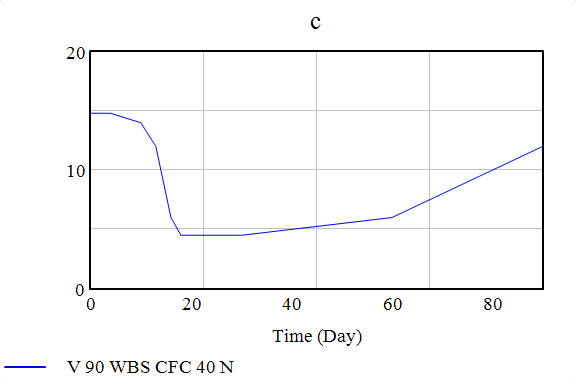 | The initial contact rate at January 10 was 14.5.^11^ From January 23 to January 29, the Level-1 emergency response was gradually launched across the country, and the contact rate sharply decreased, reaching the lowest level of 4.5 contacts per day with only family members and necessary trips. Staged work resumption started Feb 11^th^ and contact rate rose gradually reaching 13 per day within 3 months, almost returning back to the normal condition. |
| q: Quarantine rate  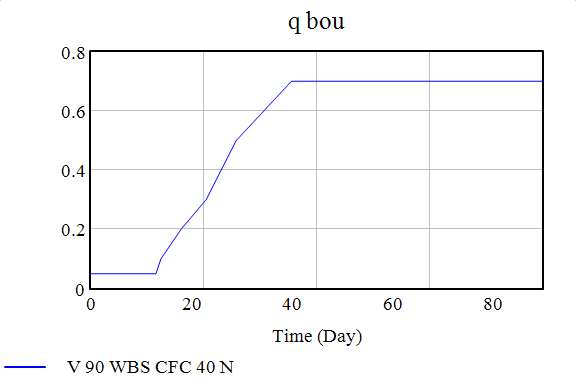 | Before the launch of the first-level emergency response on January 23, the quarantine of close contacts across the country had not been systematically carried out. Therefore, the quarantine rate was set to be only 0.05. From January 23, measures had been taken to gradually strengthen close contact tracing and quarantine.^7^ Digital technology applications for contact tracing were gradually applied. The quarantine rate of close contact increased to 0.7. |
| μ: Identification rate (IR) of fever clinic unit  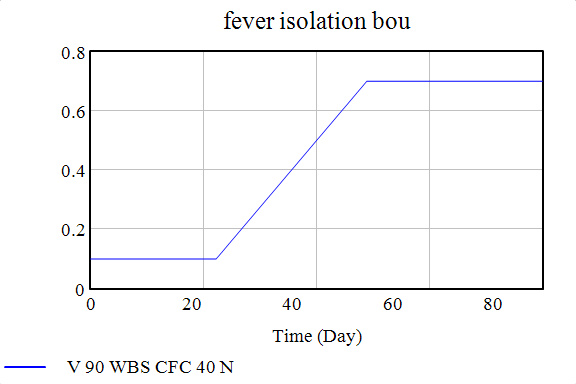 | In the beginning, there was no fever clinic unit at primary healthcare and most suspected cases with symptoms were not isolated, the IR was set to be 0.1. From February, fever clinic unit started to establish with specific focus on early identification of suspected cases. Most suspected cases were immediately isolated at fever clinic unit. But there were also cases that were not isolated after visiting fever clinic unit, causing many more people infected than otherwise. Therefore, the IR was set to increase to 0.7. |
| 1/ δ hospital acceptance rate  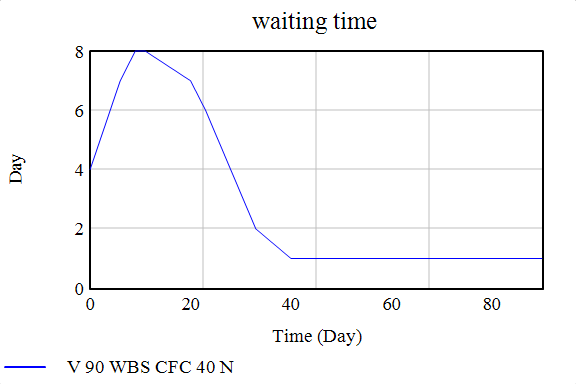 | Lack of testing capacity for coronavirus and hospital beds initially, patients had to wait several days before they can be confirmed and admitted by the hospitals.^4^ The situation became worse with the outbreak of the disease. After January 24, with supporting medical resources and investment in testing capacity, the waiting time started to drop. After February 12, all suspected and confirmed patients could be admitted to the hospitals when confirmed, the waiting time was shortened to only one day. |
| ω: Fraction of severe cases  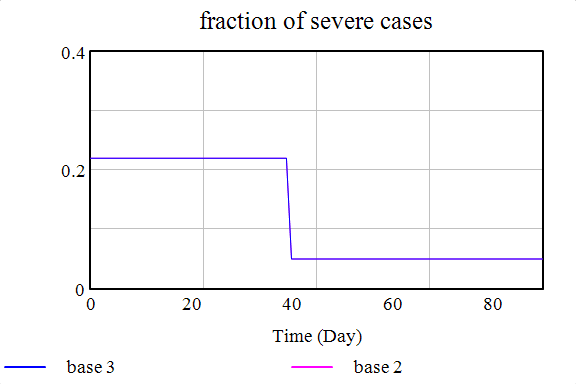 | Fraction of severe cases reduced due to the early identification and early treatment policy. Mild cases were taken good care of in makeshift hospitals, providing traditional Chinese herbal medication and other medicine and treatment when needed. In this way, most of them recovered without developing into severe cases. Therefore, the fraction of severe cases dropped from 0.22 to 0.05. |
| γ_H_: Recovery rate of mild cases  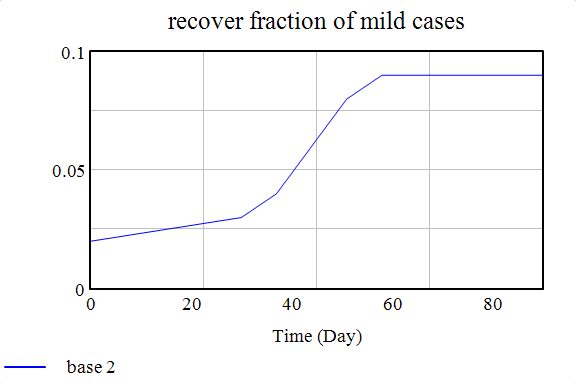 | Mild cases were admitted to the makeshift hospitals for treatment such as traditional Chinese herbal medication, antiviral therapy and oxygen inhalation to prevent their condition from turning worse. Seventh editions of the guidelines for the diagnosis and treatment of COVID-19 were published based on detailed analysis of previous cases. Therefore, the recovery rate of mild cases increased gradually from 0.02 to 0.09. |
| γ_S_: Recovery rate of severe cases  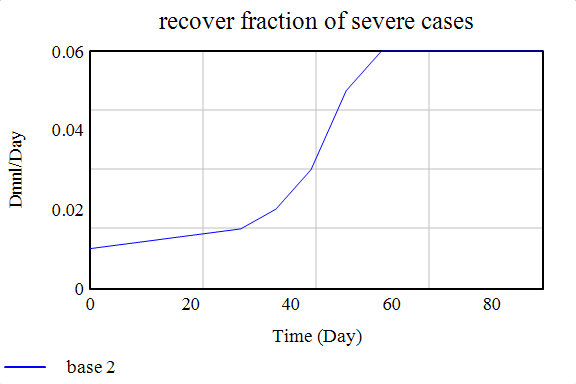 | Sever cases were admitted to designated hospitals, with experts discussing and evaluating treatment plans Recover rate of severe cases was lower than recover rate of mild cases, but it also increased as the guidelines for diagnosis and treatment of COVID-19 published. The recovery rate of severe cases increased gradually from 0.01 to 0.06. |
| α_S_: Death rate of severe cases  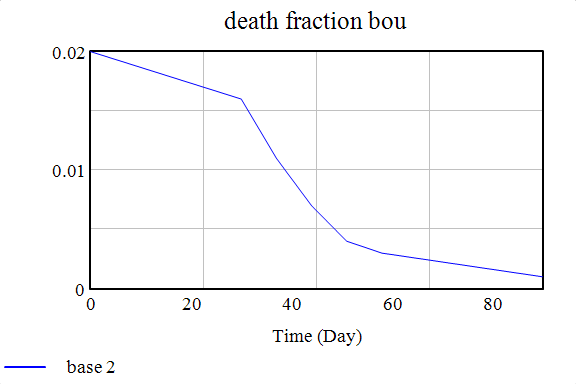 | With the development of treatment guidelines, and experts cooperation to formulate case-by-case treatment, the death rate of severe cases reduced gradually, changing from 0.02 to 0.001, which is a quite low level, since very few death occurred in the later stage of the COVID-19 outbreak. |

# Vaccination

## Vaccine roll out

Due to the successful control of the COVID-19 in China, almost the entire Chinese population is still susceptible to coronavirus. Therefore, vaccination is crucial for China to build a population immunity barrier.^12^

On Dec 5^th^ 2020, China first started vaccinating key groups,^13^ such as healthcare workers, social security workers.^14^ Mass vaccinations were implemented initially in adults aged 18 years old and above and extended to individuals aged 15-17, then 11-14, 6-11, and 4-6 step by step in the second half of 2021. The COVID-19 vaccines have been free for citizens. While vaccination is voluntary, the general public has been encouraged to get vaccinated. Most people have shown willingness to take vaccines and more than 80% population has been vaccinated before the end of 2021. Booster shots have been implemented in Dec 2021. Detailed vaccination roll-out timeline was provided in Figure 3 and the data of vaccination dose and vaccination rate in China is provided in Figure 4.

Many provinces and cities in China have started preparing for booster shot of COVID-19 vaccine

More than 2.68 billion doses of COVID-19 vaccine had been reported nationwide, and more than 1.19 billion people had been fully vaccinated.

More than 2.16 billion doses of COVID-19 vaccine had been reported nationwide, and more than 1 billion people had been fully vaccinated.

More than 1 billion doses of novel coronavirus vaccine have been reported in China

2020.7.22

Inactivated vaccines approved for emergency use in China.

Beijing biological vaccine approved for market.

2020.12.31

2020.12.19

Under the Joint Prevention and control Mechanism of the State Council, more than 1 million doses of COVID-19 vaccine have been administered to high-risk exposed people on a voluntary, informed and consenting basis since July.

2021.2.5

Sinovac biological vaccine approved for marketing.

2021.7.16

2021.12.19

2021.9.07

2021.2.25

2021.9.17

2021.9.15

2021.6.19

2021.8.26

All of our 5 technology routes have achieved clinical trial coverage, 3 inactivated vaccines have been approved for conditional marketing by SFDA, and 2 inactivated vaccines have been approved for emergency use by SFDA.

More than 2 billion dose of novel coronavirus vaccines have been reported in China.

National authorities have approved inactivated vaccines for use in people aged 3-17.

Wuhan CanSinoBIO vaccine has been approved for market.

The Novel Coronavirus Technical Guide (First edition) states that vaccination is currently for persons aged 18 and over, and recommended for persons aged 60 and over.

2021.3.29

Figure 3 The roll-out of vaccination in China

Figure 4 The number of dose and vaccination rate

Currently, China has reached more than 90% vaccination rate and has built the population immunity barrier. However, as new variants of COVID-19 continuously appeared, there existed uncertainty about the effectiveness of this barrier. In policy scenarios, we have tested various vaccination rate from 10% to 90%.

## Vaccine effectiveness

There were four types of COVID-19 vaccines that had been widely used in China. Three of them are inactivated vaccines, including vaccines produced by Sinovac Biotech Ltd., Sinopharm (China National Pharmaceutical Group Corporation), and Beijing Institute of Biological Products Co., Ltd. The fourth vaccine is the type 5 adenovirus vector vaccine produced by the CanSino Biologics company. Table 5 summarises the vaccine effectiveness which is derived from both clinical trials^15^ and vaccine effectiveness studies.^16, 17^ Based on the original trials and real-world data, infection, hospitalization, severity, and death rate were reduced after vaccination.

| **Developer** | **Type** | **Number of doses** | **Approved ages** | **Efficacy** | **Efficacy against severe disease** | **Efficacy against the symptoms** |
| --- | --- | --- | --- | --- | --- | --- |
| **Sinovac** | Corona Vac | 2,  14-28 days apart | 18+ years | 67% | \| Turkey \| 100% \| \| --- \| --- \| \| Brazil \| 87.3%(mild  and servere) \| \|  \| 100%(severe  and hospitalized) \| \| Chile \| 85%(hospitalized) \| \|  \| 89% (severe) \| \|  \| 80% (death) \| | \| Turkey \| 91.2% \| \| --- \| --- \| \| Brazil \| 50.4% \| \|  \|  \| \| Chile \| 67% \| \|  \|  \| \|  \|  \| |
| **Sinopharm** | BBIBP-CorV | 2, 14-28 days apart | 18+ years | 78.10% | 100% (mild & servere) | 72.51% |
| **Beijing Institute of biological products** | BBIBP-CorV | 2, 14-28 days apart | 18+ years | 78.10% | 99.5% (mild & servere) | 79.34% |
| **CanSino Biologics** | Ad5-nCov | 1 | 18+ years | 65.28%; 74.8%(Pakistan) | 90.98% | 65.28% |

Table 5 Vaccine effectiveness

The first three vaccines in Table 5 were inactivated vaccines which were mostly used in China. Base on the data, we set vaccination effectiveness against infection λ_1_= 80%, vaccination effectiveness against severe cases λ_2_= 90%, and vaccination effectiveness against death λ_3_= 80% for the base scenario. For new variants, the vaccination effectiveness against infection, severe cases and death were changed. As few data exists, we performed optimistic, normal and pessimistic scenarios, see section 5.4

## Waning of vaccine-induced immunity

We briefly explored the impact of the waning of vaccine effectiveness over time and the results are presented in section 5.3. Since little quantitative evidence is currently available regarding the magnitude of waning VE, we tested three scenarios with hypothetical parameter setting.

# The COVID-19 Variants

COVID-19 can acquire genetic mutations to generate new variants of the virus. Different variants come with different virulence, transmissibility, vaccine effectiveness, etc. Table 6 summarizes the key indicators of 3 major concern variants which are widely spread in the World compared with the original strain.

## Transmissibility

Compared with the original strain, 3 variants’ transmissibility are improved of significance. For the current concern variant, Omicron, there is no clear data about transmissibility, but it is more transmissible than previous variants.

## Severe illness

From Table 6, three variants can cause higher hospitalization, severity, and death rate. And there is no sufficient data about Omicron. Some news claimed that Omicron cause fewer severe cases but some others said that it had a similar severity with Delta.

## Vaccine effectiveness

As the new variants appeared, vaccine effectiveness tends to decrease because of the different mutations of each variant. Omicron has more than 30 mutations in the genes which has a high possibility of evading vaccines.

Table 6 COVID-19 Variants comparison

|  | **Original** | **Beta** | **Delta** | **Omicron** |
| --- | --- | --- | --- | --- |
| **Transmissible^18^** | 1 | 1.5 | 2 | More transmissible |
| **Hospitalization^19,20,21^** | 1 | 1.52 | 2.08 | 1.48 |
| **Severity^19,20,22^** | 1 | 1.89 | 3.35 | Insufficient data  (no signal supports a difference compared to Delta) |
| **Death rate^19,20^** | 1 | 1.51 | 1.33 | Not clear |
| **Vaccine efficacy^18,19,23^** |  | 75% | 59% | Not clear  Capable of at least partially evading vaccines |

# Model validation

## Data

The base model is simulated from 0-90 days, corresponding to the outbreak of COVID-19 in China from Jan 10th 2020 to Apr 10th 2020. Historical data of COVID-19 transmission in China were used to validate the model. The daily reports published by the National Health Commission of the People’s republic of China were the source of the historical data, details listed in Table 7.

Table 7 Data source for validation

| **Data** | **Description** | **Reference** |
| --- | --- | --- |
| **New confirmed cases** | Daily number of confirmed patients admitted to hospital. | Daily report from National Health Commission of the People’s republic of China^2^ |
| **Cumulative confirmed cases** | Time-to-date total confirmed cases. |  |
| **Recovery from hospital** | Daily number of patients released from hospital, the sum of recover from mild cases and recover from severe cases. |  |
| **Cumulative recover** | Time-to-date total recovered cases, the sum of cumulative recover from mild cases and severe cases. |  |
| **Death from severe cases** | Daily number of patients died from hospital. |  |
| **Cumulative death** | Time-to-date total death from hospital. |  |
| **Hospitalized population** | The current patients in hospital. |  |
| **Severe cases** | The current severe cases in hospital. |  |

## Simulation results and historical data

Figure 5 The simulation result and historical data

Simulation results showed that accumulative variables such as the number of cumulative confirmed cases, the number of cumulative recoveries, and the number of cumulative death fit well with historical data. Daily new confirmed cases, new recover and new death had more fluctuation and uncertainty, therefore, did not fit point-to-point. However, the simulation result did reflect the general trajectory well, adding confidence to the model.

Table 8 compares the simulated results with historical data. The peak values of the hospitalized population and severe cases as well as the peak dates. The simulated peak date of hospitalized cases and severe cases is 1 day ahead of and 1 day later than the historical data respectively, and its peak value shows −11.66% and 5.97% difference from the historical data. For cumulative confirmed cases and cumulative deaths on Apr. 10th, the differences were 5.87% and -0.89%, respectively.

Table 8 Comparing simulated results with historical data

|  | Simulated | Historical | Difference (%) |
| --- | --- | --- | --- |
| Hospitalized population peak value | 51,251 | 58,016 | -11.66% |
| Hospitalized population peak date | Feb 18th | Feb 17th | -1 days |
| Severe cases peak value | 11,263 | 11,977 | -5.97% |
| Severe cases peak date | Feb 19th | Feb 18th | 1 days |
| Cumulative confirmed cases on Apr.10th | 86,765 | 81,953 | 5.87% |
| Cumulative deaths on Apr.10th | 3,309 | 3,339 | -0.89% |

## 4.3 Sensitive tests

Due to the uncertainty of the parameters, we performed sensitive tests for some of the key variables such as transmission probability, incubation period and some initial values of population groups. Even though some variables showed higher sensitivity than other variables, the behavior pattern remained unchanged, adding confidence to the model. Details please refer to Figure 6-Figure 10.

**Test variable: transmission probability β**

Parater Setting:

| transmission probability | Base | Sensitive test | | | |
| --- | --- | --- | --- | --- | --- |
|  |  | min | max | mean | SD |
| β | 0.038 | 0.0342 | 0.0418 | 0.038 | 0.037 |


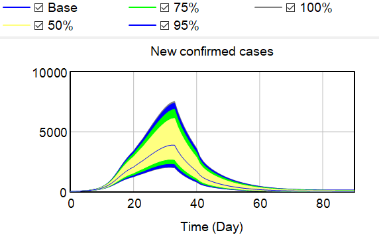

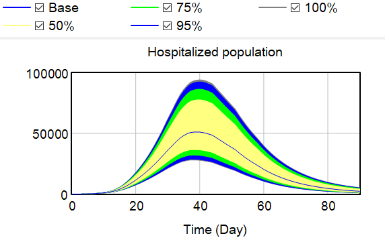

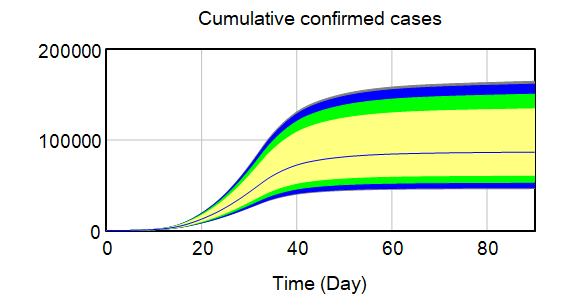

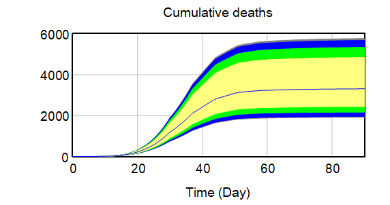


Figure 6 Sensitivity tests for transimission probability

It was clear that the transmission probability had high sensitivity: 10% increase or decrease caused the simulation results to vary over large range. However, the behavior pattern remained the same.

**Test variable: initial exposed population E0**

Parater Setting:

| initial exposed population | Base | Sensitive test | | | |
| --- | --- | --- | --- | --- | --- |
|  |  | min | max | mean | SD |
| E0 | 450 | 405 | 495 | 450 | 200 |


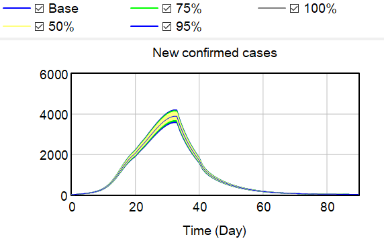

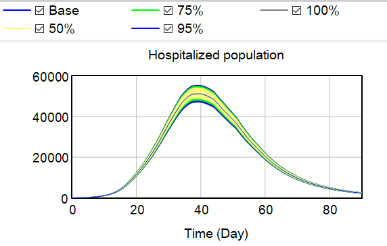

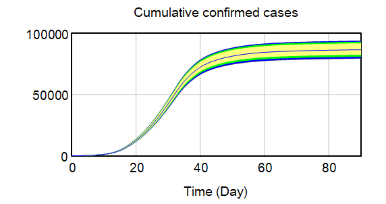

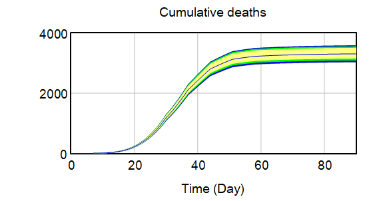


Figure 7 Sensitivity tests for initial exposed population

The initial level of exposed population had little sensitivity over model behavior. 10% of increase or decrease generated only slight increase or decrease in simulation result.

**Variable: initial infected population I0**

Parater Setting:

| initial infected population | Base | Sensitive test | | | |
| --- | --- | --- | --- | --- | --- |
|  |  | min | max | mean | SD |
| I0 | 100 | 90 | 110 | 100 | 50 |


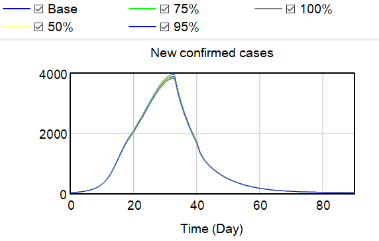

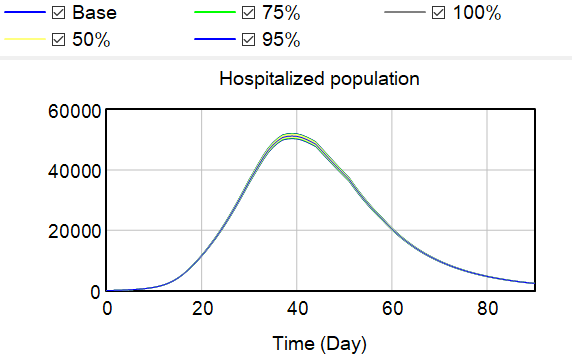

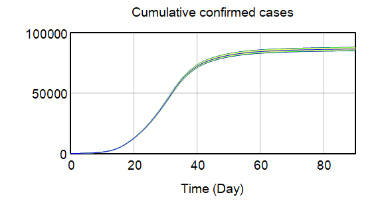

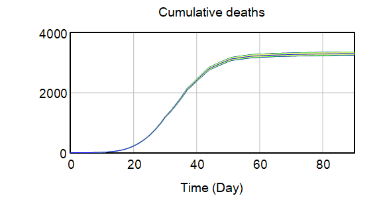


Figure 8 Sensitivity tests for initial infected population

The initial level of infected population had almost no sensitivity over model behavior. 10% of increase or decrease almost had no impact on the simulation result.

**Test variable: Infectiousness in incubation period θ**

| Infectiousness in incubation period | Base | Sensitive test | | | |
| --- | --- | --- | --- | --- | --- |
|  |  | min | max | mean | SD |
| θ | 0.5 | 0.45 | 0.55 | 0.5 | 0.3 |


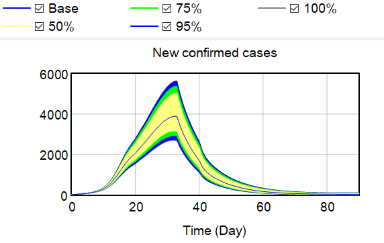

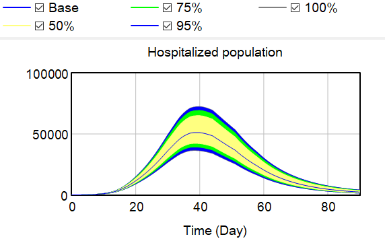

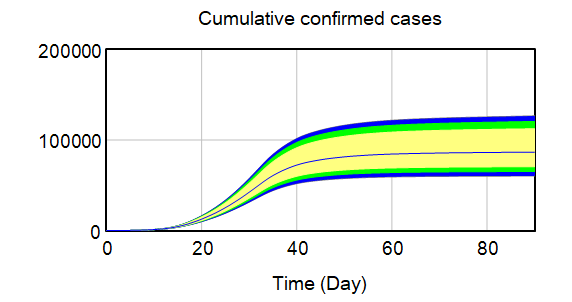

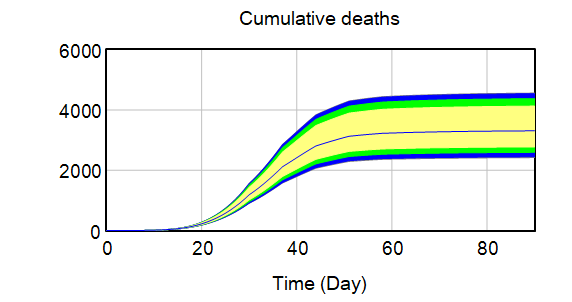


Figure 9 Sensitivity tests for infectiousness in incubation period

The infectiousness in incubation period had high sensitivity: 10% increase or decrease caused the simulation results to change around 50%. However, the behavior pattern remained the same.

**Test variable: incubation period 1/σ**

Parameter setting:

| incubation period | Base | Sensitive test | | | |
| --- | --- | --- | --- | --- | --- |
|  |  | min | max | mean | SD |
| 1/σ | 5.2 | 4.68 | 5.72 | 5.2 | 3 |

Figure 10 Sensitivity tests for infectiousness in incubation period


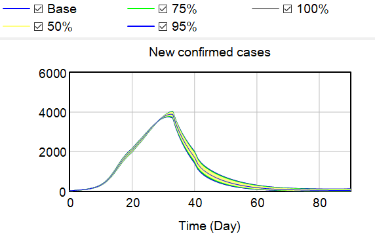

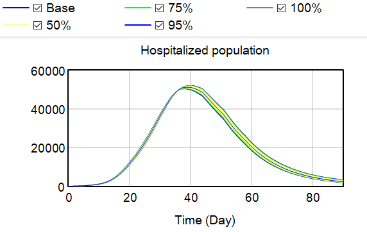

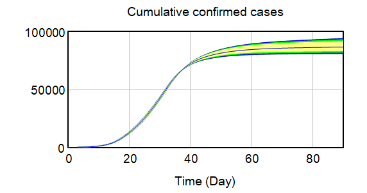

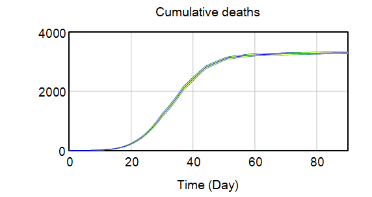


The incubation period had low sensitivity: 10% increase or decrease didn’t change the simulation results much.

# Forward projection

## Severe cases under combined policy

Here, we presented the simulation results for severe cases under combined policy. When vaccination rate was lower than 30%, severe cases peaked at several million no matter with border screening or not. Severe cases stabilized when vaccination rate reached 70%, at a lower level with border screening. Higher fever clinic unit identification rate (IR) was most effective when vaccination rate was low. When most people were vaccinated, high IR rate had little impact. For the new variant with high transmission probability and lower vaccination effectiveness against infection, the severe case didn’t stabilize until vaccination rate reached 90%.


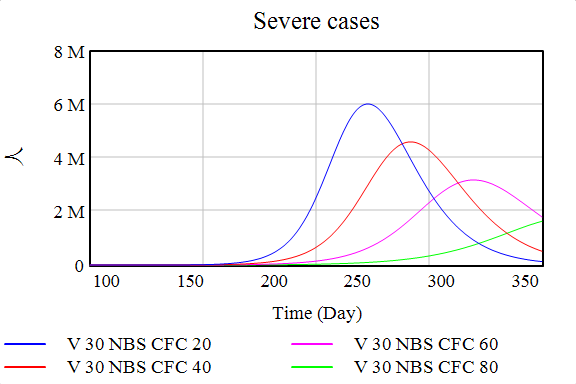


**30% Vaccination**


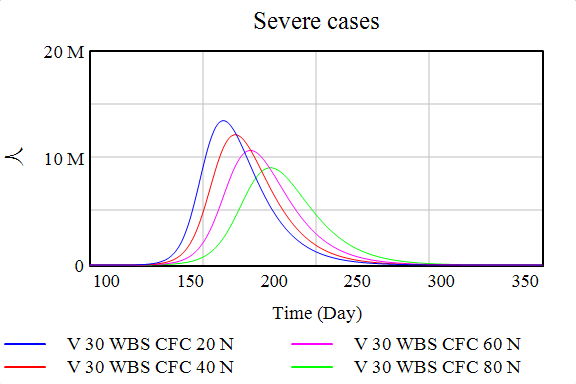

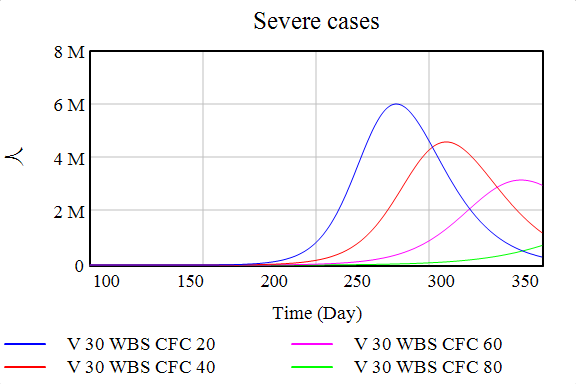


**30% Vaccination**

**30% Vaccination**


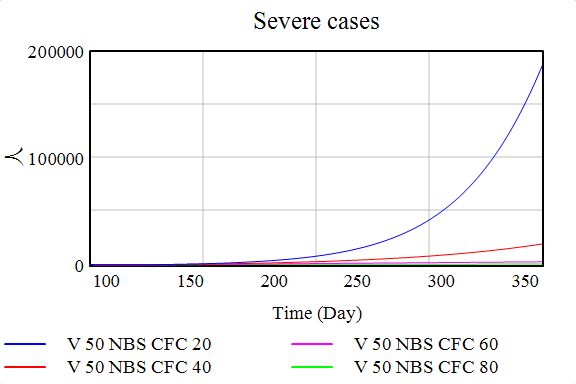

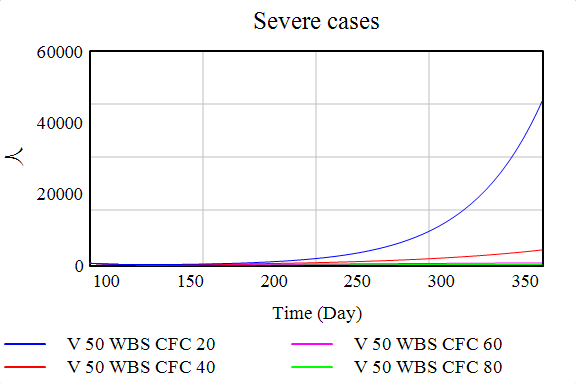

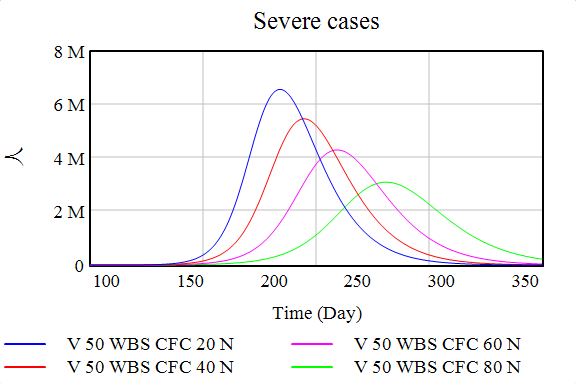


**50% Vaccination**

**50% Vaccination**

**50% Vaccination**


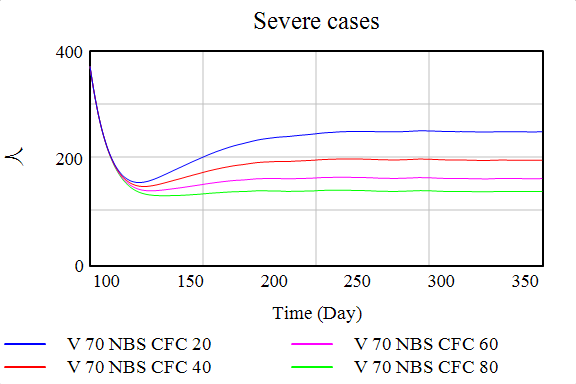

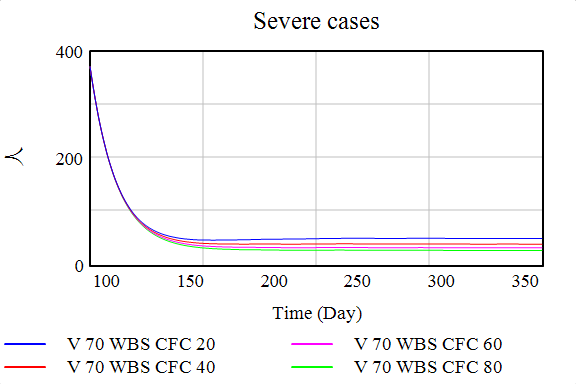

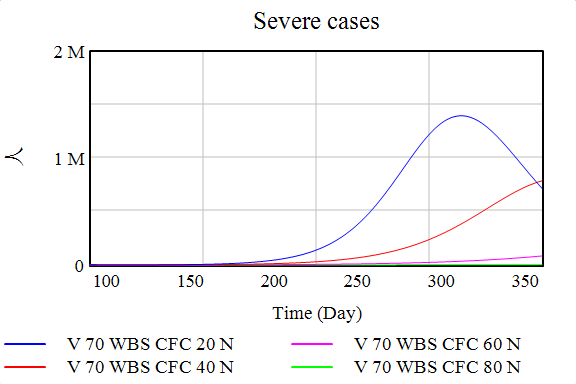


**70% Vaccination**

**70% Vaccination**

**70% Vaccination**


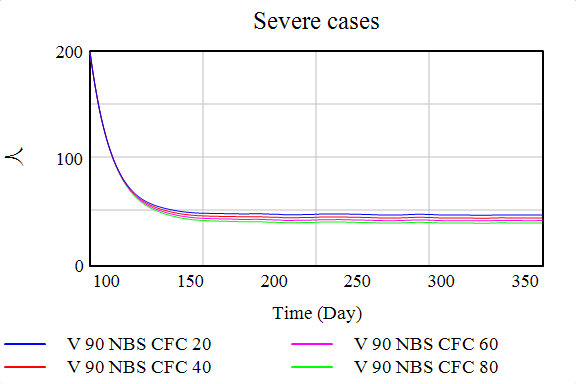

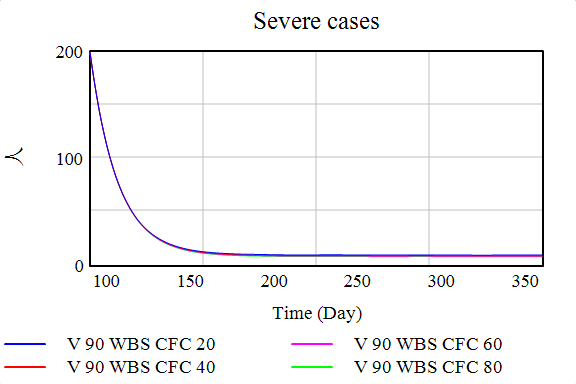


**90% Vaccination**

**90% Vaccination**


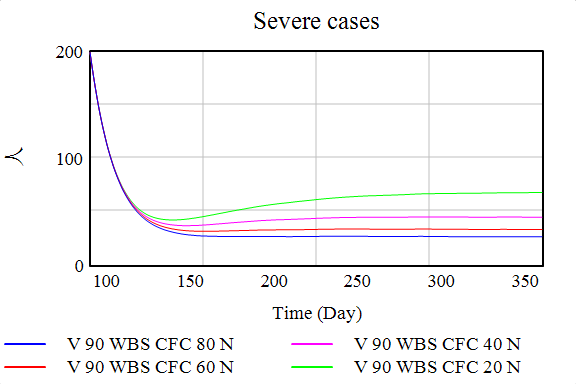


**90% Vaccination**


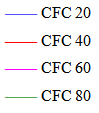


IR 20%

IR 40%

IR 60%

IR 80%

1. (b) (c)

Figure 11 Simulation result for severe cases under combined policy: (a)without border screening, (b) with border screening and (c) the scenario of new variant with 30% higher infectivity and lower vaccination effectiveness against infectious, at 70% compared with 80% with the normal variant.

## Cumulative deaths under combined policy

Here, we presented the simulation results for cumulative deaths under combined policy. When vaccination rate was lower than 30%, cumulative deaths would peak at several hundred thousands no matter with border screening or not. Cumulative deaths stabilized when vaccination rate reached 70%, at a lower level with border screening. Higher fever clinic unit identification rate (IR) was most effective when vaccination rate was low. When most people were vaccinated, high IR rate had little impact. For the new variant with high transmission probability and lower vaccination effectiveness against infection, the cumulative death didn’t stabilize until vaccination rate reached 90%.


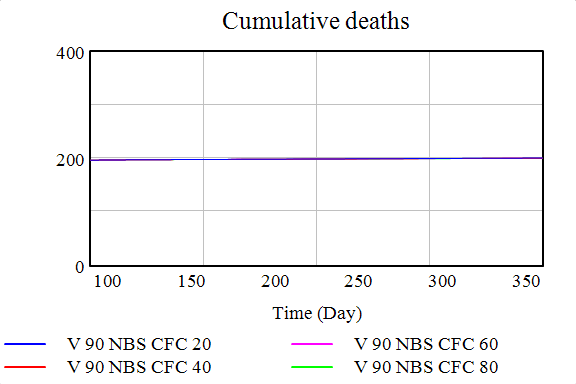

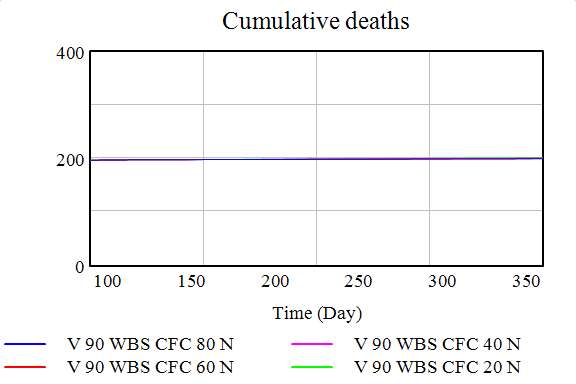

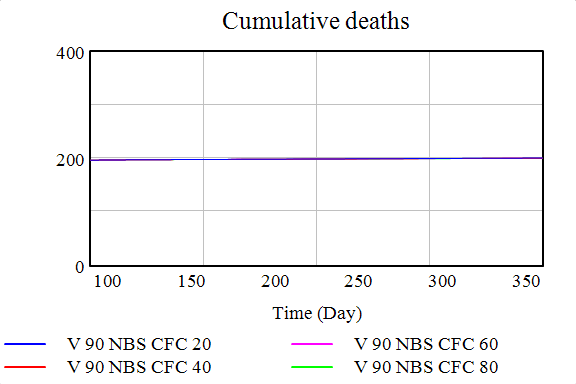


**90% Vaccination**

**90% Vaccination**

**90% Vaccination**


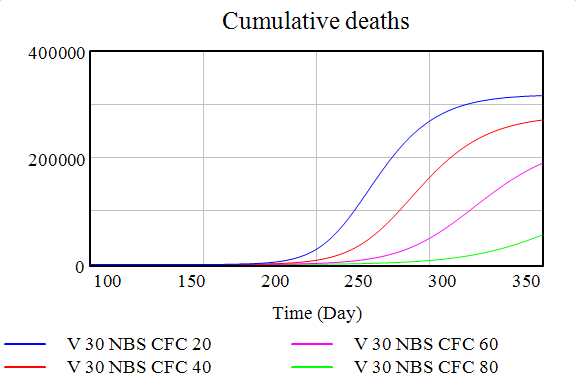


**30% Vaccination**


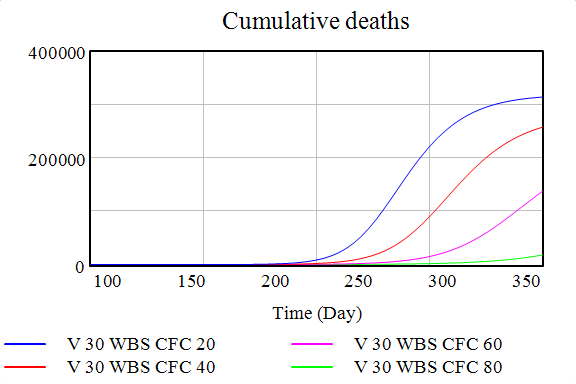


**30% Vaccination**


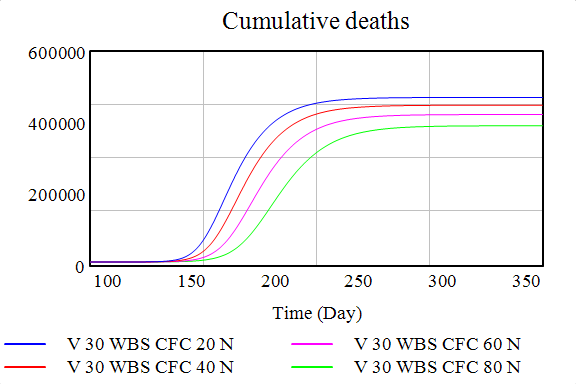


**30% Vaccination**


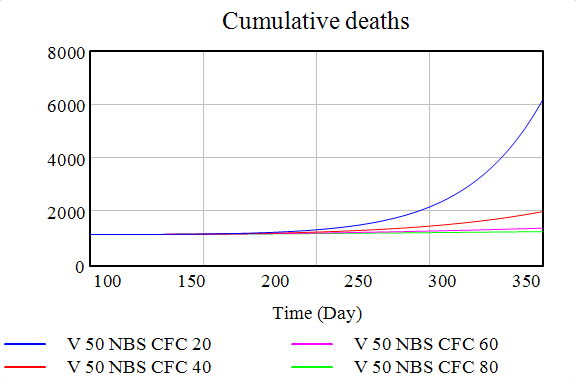

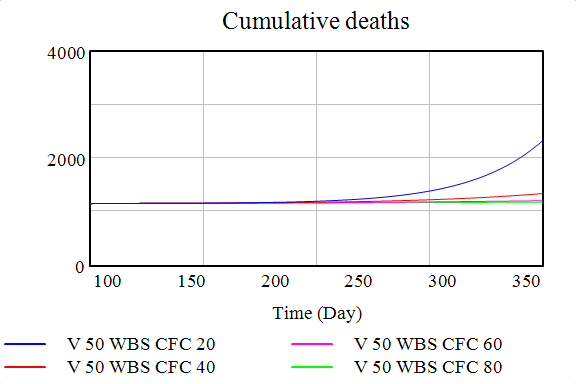

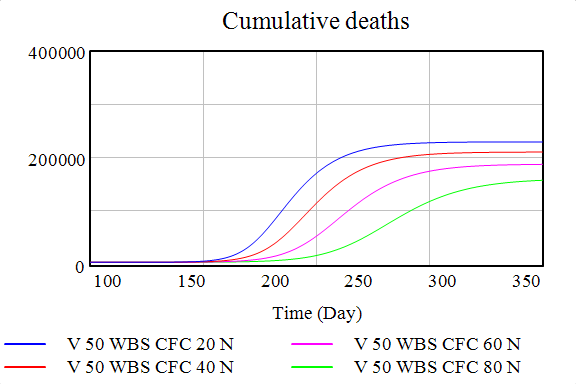


**50% Vaccination**

**50% Vaccination**

**50% Vaccination**


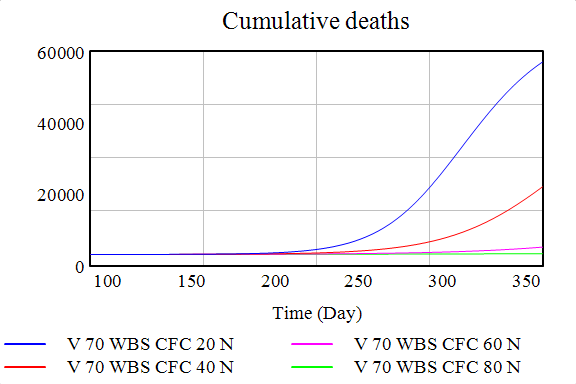

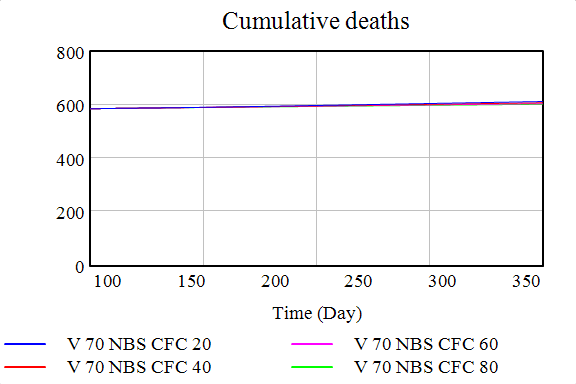


**70% Vaccination**


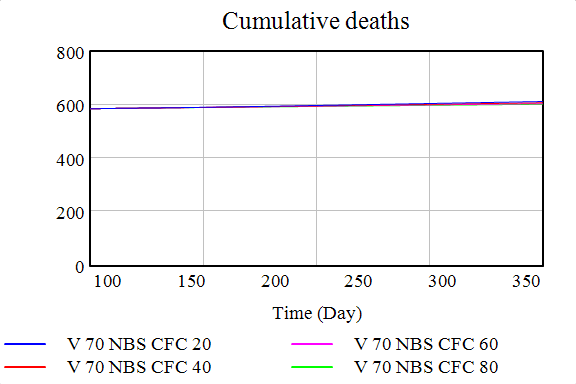


**70% Vaccination**

**70% Vaccination**


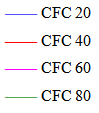


IR 20%

IR 40%

IR 60%

IR 80%

1. (b) (c)

Figure 12 Simulation result for cumulative deaths under combined policy: (a)without border screening, (b) with border screening and (c) the scenario of new variant with 30% higher infectivity and lower vaccination effectiveness against infectious, at 70% compared with 80% with the normal variant.

## Waning effects of vaccine-induced immunity

Many data showed that there is waning effect of vaccine-induced immunity. However, detailed data about the waning effect of vaccination is not identified. Using the model, we tested the three scenarios that vaccination-induced immunity waning effect, starting two months after vaccination and in four months time, reduce the effectiveness of vaccination to 30%, 50%, and 70%. Here, the effectiveness of vaccine applies to effectiveness of vaccination against infection, against severe cases and against death, as shown in Figure 13. We use 70% vaccination, with board screening, and 80% effectiveness of fever clinic unit as the case for investigation.


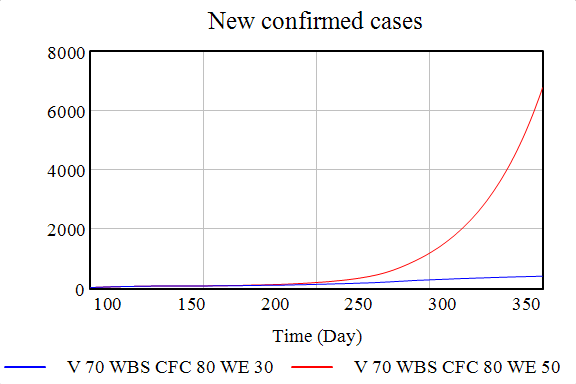

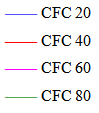

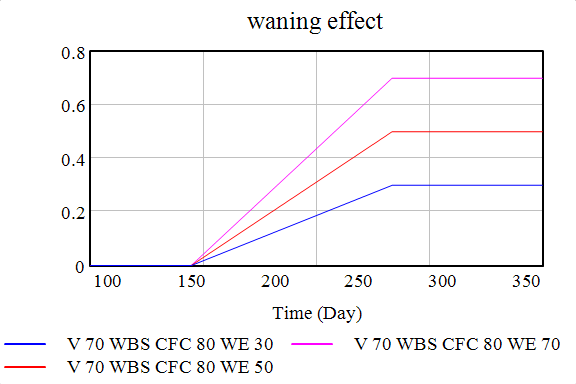

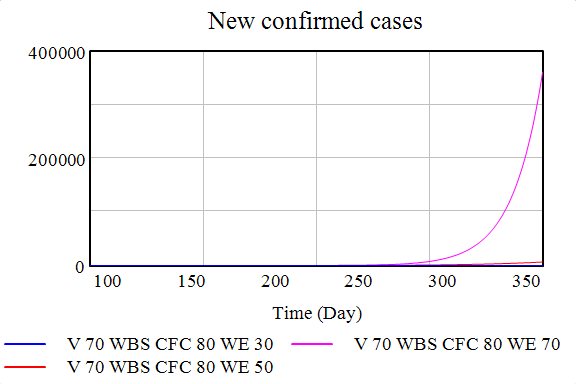


waning effect 30%

waning effect 50%

waning effect 70%


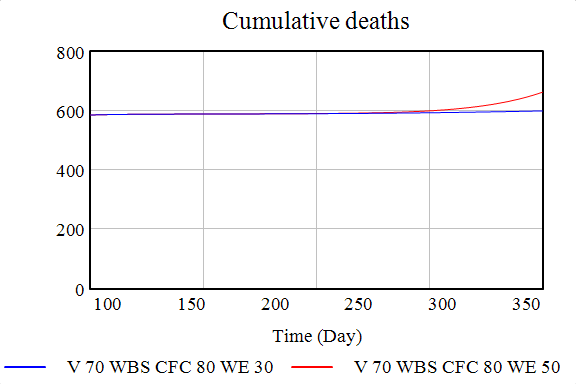

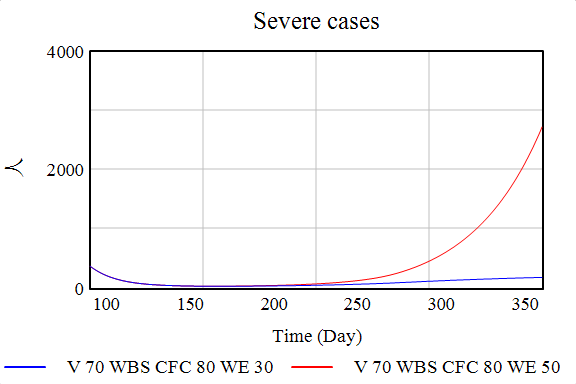

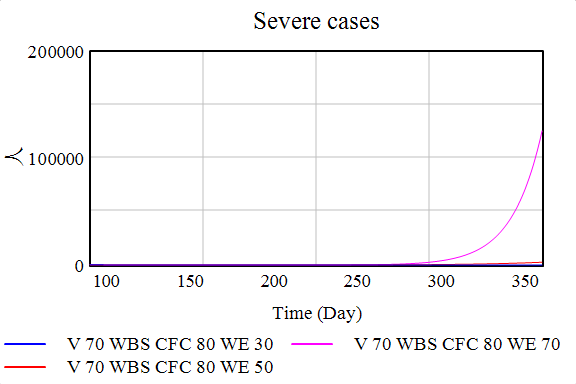

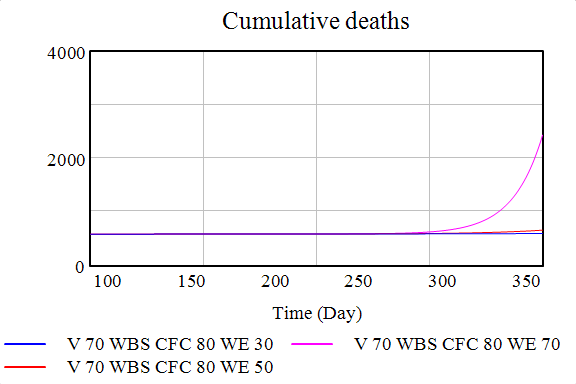


Figure 13 Simulation result of waning effect

The simulation results showed that higher waning effect, 50% and 70% caused rebound in new confirmed cases, severe cases and cumulative deaths as well. But low waning effect, such as 30% didn’t cause a large increase in these variables at the end of simulation.

## COVID-19 variants scenarios

First of all, we performed three types of new variants, one with double infectivity, one with double fraction of severe cases and one with double death rate under the condition that 70% vaccination rate, 80% fever clinic unit IR and border screening. Results are shown in Figure 14.

| Double infectivity | | |
| --- | --- | --- |
|  |  |  |
| Double fraction of severe cases | | |
|  |  |  |
| Double death rate | | |
|  |  |  |

Figure 14 Simulation result of new variant with doubling infectivity, severity and death rate respectively

Simulation results have shown that if the new variant doubled infectivity, even with 70% vaccination rate and high IR at fever clinic unit, the new confirmed cases increase exponentially. Moreover, even the severity remained unchanged, due to more confirmed cases, the number of severe cases also increased. Due to the protection from vaccination, severe cases didn’t increase as much as new confirmed cases and the cumulative death didn’t increase much. This is similar to the case of Omicron variant that we are facing: a new peak of new confirmed cases, more severe cases, and a bit more death. Vaccination is an important way to protect people from severe cases and death. The new variant didn’t increase infectivity, but doubled severity or death rate, due to the protection from vaccination, few people were infected, leading to almost no change in the number of severe cases and cumulative deaths. Above all, vaccination is of great importance in protecting people against new variants, either from getting infected or from getting severe illness or death.

Secondly, we performed three scenarios changing the vaccination effectiveness against the new variants. As there is little specific data on delta and even less on omicron, we assume three scenarios: optimistic scenario, normal scenario and pessimistic scenario with the following parameter settings in table 8:

Table 8 Data source for validation

|  | Base scenario | optimistic | normal | pessimistic |
| --- | --- | --- | --- | --- |
| VE against infection | 0.8 | 0.7 | 0.6 | 0.5 |
| VE against severe cases | 0.9 | 0.8 | 0.7 | 0.6 |
| VE against death | 0.8 | 0.7 | 0.6 | 0.5 |

From figure 15, we can see that in optimistic case, new confirmed cases, severe cases stabilized at low level and almost no death occurred as cumulative death also stayed unchanged. But in the normal scenario, new confirmed cases stabilized at higher level, with slight increase in severe cases and cumulative death, meaning new death occurred. In the pessimistic scenario, new confirmed cases and severe cases increase exponentially and cumulative deaths showed clear increase. It was obvious that under the condition vaccination effectiveness against infection, severity and death all reduced 30%, we would face rebound of the epidemic again.

| optimistic | | |
| --- | --- | --- |
|  |  |  |
| normal | | |
|  |  |  |
| pessimistic | | |
|  |  |  |

Figure 15 Simulation result of new variant considering the reduction of vaccination effectiveness

# Reference

1. National Bureau of Statistics of China. China statistical year book 2021. <http://www.stats.gov.cn.proxy.www.stats.gov.cn/tjsj/ndsj/2021/indexch.htm>. (accessed 04 Jan 2020)

2. National Health Commission of People’s Republic of China. The epidemic report. (2021). <http://www.nhc.gov.cn/xcs/yqtb/list_gzbd_29.shtml>. (accessed 04 Jan 2022)

3. Imperial CollegeCOVID-19 Response Team: Spiral: Report 9: Impact of nonpharmaceutical interventions (NPIs) to reduce COVID-19 mortality and healthcare demand. (2020). <https://spiral.imperial.ac.uk:8443/handle/10044/1/77482>. (accessed 15 October 2020)

4. Li Q, Guan X, Wu P, Wang X, Zhou L, Tong Y, et al. Early transmission dynamics in Wuhan, China, of novel coronavirus-infected pneumonia. *New Engl J Med.* (2020) 382:1199–207.

5. Zhang J, Litvinova M, Wang W, Wang Y, Deng X, Chen X, et al. Evolving epidemiology and transmission dynamics of coronavirus disease 2019 outside Hubei province, China: a descriptive and modelling study. *Lancet Infect Dis*. (2020) 20:793–802.

6. Ferretti L, Wymant C, Kendall M, et al. Quantifying SARS- CoV-2 transmission suggests epidemic control with digital contact tracing. *Science*. (2020) 368:eabb6936.

7. Shanghai Municipal Health Commission. What are the precautions for those returning to Shanghai to stay at home for 14 days? (2020). <http://wsjkw.sh.gov.cn/xwfb/20200204/db01b711b12040c4bbba663289ea00f7.html>. (Accessed 15 Oct 2020)

8. Prem K, Liu Y, Russell TW, Kucharski AJ, Hellewell J. The effect of control strategies to reduce social mixing on outcomes of the COVID-19 epidemic in Wuhan, China: a modelling study. *Lancet Public Health*. (2020) 5:E261-70.

9. Qian Y, Xie W, Zhao J, Xue M, Liu S, Wang L, Li W, Dai L, Cai Y. Investigating the effectiveness of re-opening policies before vaccination during a pandemic: SD modelling research based on COVID-19 in Wuhan. *BMC Public Health*. (2021) 21:1638.

10. Hubei Daily. Ensuring that suspected and confirmed cases are admitted to medical observation ASAP. (2020). <https://epaper.hubeidaily.net/pc/column/202002/06/node_01.html> . (accessed 19 Aug 2021)

11. Tang B, Wang X, Li Q, Bragazzi NL, Tang S, Xiao Y, Wu J. Estimation of the transmission risk of the 2019-nCoV and its implication for public health interventions. *J Clin Med*. (2020) 9:462.

12. Zheng, W., Yan, X., Zhao, Z. *et al.* COVID-19 vaccination program in the mainland of China: a subnational descriptive analysis on target population size and current progress. *Infect Dis Poverty*. (2021) 10:1-10.

13. China Daily. Q&A on COVID-19 vaccination in China. (2021). <https://covid-19.chinadaily.com.cn/a/202101/07/WS5ff6811aa31024ad0baa1194_1.html>. (accessed 04 Jan 2022)

14. COVID-19 Vaccine Technical Working Group. Technical Vaccination Recommendations for COVID-19 Vaccines in China (First Edition)[J]. *China CDC Weekly.* (2021) 3:459-61.

15. Liao C, Wang Z, et al. Progress post-marketing safety and efficacy studies of COVID-19 vaccine. *Journal of Jinan University (Natural Science & Medicine Edition)*. (2021) 42:547-56.

16. Forward The Economist. Chinese COVID-19 vaccine has achieved multi-route research and development, and a comprehensive comparison of 5 vaccines that have been on the market. (2021). <https://www.qianzhan.com/analyst/detail/220/210607-1bba6e9c.html>. (accessed 04 Jan 2022)

17. Xinhua Net. Chile updates Coxing vaccine protection effect data. (2021). <http://www.xinhuanet.com/world/2021-05/18/c_1127462029.htm>. (accessed 04 Jan 2022)

18. Live Science. Coronavirus variants: Facts about omicron, delta and other COVID-19 mutants. (2021). <https://www.livescience.com/coronavirus-variants.html>. (accessed 04 Jan 2022)

19. CDC. Omicron Variant: What You Need to Know. (2021). <https://www.cdc.gov/coronavirus/2019-ncov/variants/omicron-variant.html>. (accessed 04 Jan 2022)

20. RACGP. Study suggests Delta more than doubles death risk. (2021). <https://www1.racgp.org.au/newsgp/clinical/study-suggests-delta-more-than-doubles-death-risk>. (accessed 04 Jan 2022)

21. Nature. How severe are Omicron infections? (2021). <https://www.nature.com/articles/d41586-021-03794-8>. (accessed 04 Jan 2022)

22. UK Health Security Agency. Risk assessment for SARS-CoV-2 variant: Omicron VOC-21NOV-01 (B.1.1.529). (2021). <https://assets.publishing.service.gov.uk/government/uploads/system/uploads/attachment_data/file/1041896/15-december-2021-risk-assessment-for-SARS_Omicron_VOC-21NOV-01_B.1.1.529.pdf>. (accessed 04 Jan 2022)

23. Li XN, Huang Y, Wang W, et al. Effectiveness of inactivated SARS-CoV-2 vaccines against the Delta variant infection in Guangzhou: a test-negative case–control real-world study. *Emerging microbes & infections.* (2021) *10*:1751-1759.
